# Auditing sex- and gender-based medicine (SGBM) content in medical school curriculum: a student scholar model

**DOI:** 10.1186/s13293-016-0102-x

**Published:** 2016-10-14

**Authors:** Michael M. Song, Betsy G. Jones, Robert A. Casanova

**Affiliations:** 1 School of Medicine and Graduate School of Biomedical Sciences, School of Medicine Cancer Center, Laura W. Bush Institute for Women’s Health, Texas Tech University Health Sciences Center, 3601 4th St. Stop 9224, Lubbock, TX 79430 USA; 2 Department of Medical Education, School of Medicine, Laura W. Bush Institute for Women’s Health, Texas Tech University Health Sciences Center, 3601 4th St. Stop 6525, Lubbock, TX 79430-0002 USA; 3 Department of Obstetrics and Gynecology, School of Medicine, Laura W. Bush Institute for Women’s Health, Texas Tech University Health Sciences Center, 3601 4th St. Stop 8326, Lubbock, TX 79430 USA

**Keywords:** Sex- and gender-based medicine (SGBM), Medical school curriculum evaluation, SGBM curriculum integration

## Abstract

**Background:**

Sex- and gender-based medicine (SGBM) aims to (1) delineate and investigate sex- and gender-based differences in health, disease, and response to treatment and (2) apply that knowledge to clinical care to improve the health of both women and men. However, the integration of SGBM into medical school curricula is often haphazard and poorly defined; schools often do not know the current status of SGBM content in their curricula, even if they are committed to addressing gaps and improving SGBM delivery. Therefore, complete auditing and accounting of SGBM content in the existing medical school curriculum is necessary to determine the baseline status and prepare for successful integration of SGBM content into that curriculum.

**Methods:**

A review of course syllabi and lecture objectives as well as a targeted data analysis of the Curriculum Management and Information Tool (CurrMIT) were completed prior to a real-time curriculum audit. Subsequently, six “student scholars,” three first-year and three second-year medical students, were recruited and trained to audit the first 2 years of the medical school curriculum for SGBM content, thus completing an audit for both of the pre-clinical years simultaneously. A qualitative analysis and a post-audit comparative analysis were completed to assess the level of SGBM instruction at our institution.

**Results:**

The review of syllabi and the CurrMIT data analysis did not generate a meaningful catalogue of SGBM content in the curriculum; most of the content identified specifically targeted women’s or men’s health topics and not sex- or gender-based differences. The real-time student audit of the existing curriculum at Texas Tech revealed that most of the SGBM material was focused on the physiological/anatomical sex differences or gender differences in disease prevalence, with minimal coverage of sex- or gender-based differences in diagnosis, prognosis, treatment, and outcomes.

**Conclusions:**

The real-time student scholar audit was effective in identifying SGBM content in the existing medical school curriculum that was not possible with a retrospective review of course syllabi and lecture objectives or curriculum databases such as the CurrMIT. The audit results revealed the need for improved efforts to teach SGBM topics in our school’s pre-clinical curriculum.

**Electronic supplementary material:**

The online version of this article (doi:10.1186/s13293-016-0102-x) contains supplementary material, which is available to authorized users.

## Background

An increasing body of evidence indicates that sex or gender differences in diseases common to both women and men can result in variations in outcomes stemming from dissimilarities in epidemiology, pathology, presentation, and treatments [1]. Therefore, sex and gender are essential elements of individualized medicine [2, 3]. Sex- and gender-based medicine (SGBM) focuses on delineating and defining sex- or gender-based differences through research and applying the results of that research to clinical care that improves the health and outcomes of both women and men [4, 5]. Although a 2001 Institute of Medicine report emphasized the importance of sex- and gender-based medicine [6], a 2003–2004 online curriculum review indicated that less than 30 % of medical schools had SGBM topics in their curricula [7] and a 2004–2005 medical student survey revealed only brief-to-moderate coverage of SGBM content [8]. Furthermore, in a survey of allopathic and osteopathic medical schools completed in 2011, 70 % of the responding institutions indicated that they did not have a formal, integrated SGBM curriculum (M.J., 2016, unpublished data).

Effective accounting and mapping of curricular content is essential to the effective development of any integrated curriculum [9, 10]. Curriculum mapping is often completed from the perspective of instructors [11, 12], utilizing lecture objectives, course syllabi, or centralized electronic curriculum databases such as the Curriculum Management and Information Tool (CurrMIT) developed by the Association of American Medical Colleges (AAMC). As for curriculum mapping from the perspective of learners in healthcare programs, Plaza et al. reported utilizing students to map pharmacy school curriculum through retrospective portfolio reviews [13]. Wong and Roberts engaged residents and class representatives to update the topics on an internal medicine didactics curriculum, starting with retrospective data as a baseline, before aligning the curriculum with learning objectives from the in-training exam. This approach allowed them to cover examination topics without duplication and omissions [14].

As a part of a larger institutional initiative to fully integrate SGBM content into medical school curriculum at the Texas Tech University Health Sciences Center (TTUHSC) School of Medicine (SOM), a complete needs assessment was necessary to accurately characterize the status of SGBM content in the existing first- and second-year, pre-clinical medical curriculum. Since SGBM content is difficult to categorize and currently not classified by specific Medical Subject Heading (MeSH) terms, we expected SGBM topics to be ambiguously cataloged at best and, therefore, poorly represented in electronic databases. Therefore, in addition to a review of published course syllabi and a retrospective analysis of CurrMIT curriculum data, an in-person real-time audit would likely be necessary to effectively account for all SGBM contents in the existing medical school curriculum. The overarching goal of this study was to evaluate the status of SGBM content in pre-clinical curriculum at Texas Tech, identify major gaps, and identify approaches and methods that would facilitate the integration of SGBM content.

## Methods

### Organization

The TTUHSC is a multi-campus, multi-disciplinary institution in West Texas; its Schools of Medicine, Nursing, Pharmacy, Health Professions, and Public Health and the Graduate School of Biomedical Sciences are responsible for providing health care to 108 counties in the western half of Texas as well as surrounding counties in Oklahoma and New Mexico.

The medical school curriculum audit program was conceptualized and designed by Marjorie Jenkins, M.D., for the Laura W. Bush Institute for Women’s Health, a TTUHSC institute whose work focuses on research, teaching, and outreach related to women’s health and SGBM. The work of the audit was carried out by the Gender-Specific and Women’s Health Committee established at Texas Tech in 2010, which later became known as the Sex- and Gender-Based Medicine (SGBM) Committee. The committee included multiple stakeholders, including basic science and clinical faculty from multiple disciplines and curriculum deans (Table 1).

**Table 1:** Membership composition of the TTUHSC SOM SGBM Curriculum Committee

Name	Degree	Department
Marjorie Jenkins	M.D.	Internal Medicine–Gender-Specific Women’s Health
Joanna Wilson	D.O.	Internal Medicine–Gender-Specific Women’s Health
Roberto Casanova	M.D.	Obstetrics and Gynecology; Asst. Dean of Clinical Sciences Curriculum
Bradley Miller	M.D./Ph.D.	Pathology–Neuropathology
Betsy Jones	Ed.D.	Medical Education
Leigh Johnson	Ph.D.	Family and Community Medicine (qualitative analysis)
Simon Williams	Ph.D.	Medical Education; Associate Dean for Medical Curriculum
3 MS-1 student scholars		School of Medicine
3 MS-2 student scholars		School of Medicine
1 summer student scholar		School of Medicine

The committee was charged with assessing medical student knowledge, attitudes, and competencies for SGBM. This assessment was to serve as the basis for the integration of SGBM content into the school’s curriculum. The initial goal was to complete a comprehensive needs assessment, both general and targeted, with the ultimate goal of establishing a fully integrated, evidence-based SGBM curriculum for the medical school.

### Retrospective review of course syllabi and lecture objectives

In 2009, the syllabus for each pre-clinical course, along with a complete set of lecture slides with session objectives, was collected from Blackboard, an online learning environment system (Blackboard, Inc., Washington, D.C.) used by the School of Medicine. The faculty members on the TTUHSC SGBM Committee retrospectively reviewed the syllabi and lecture objectives for SGBM content.

### Targeted review of Texas Tech CurrMIT database

A search of CurrMIT, an online centralized curriculum database from the AAMC Division of Medical Education, was carried out in 2009 to identify SGBM content already present in the medical school curriculum at the TTUHSC. The range of data queried was for the graduating classes of 2009, 2010, 2011, and 2012. As no SGBM-specific category was available, the search was narrowed by focusing on “women’s health” and “hot topics,” which include a list of important and timely topics as defined by the Liaison Committee on Medical Education (LCME). The extracted CurrMIT data were reviewed for SGBM content by multiple faculty members on the Texas Tech SGBM Committee.

### Recruitment and training of “student scholars”

With the initial retrospective reviews of course syllabi, lecture objectives, and CurrMIT database analysis completed, a proposal for a real-time student scholar model audit for SGBM content in the pre-clinical years was approved by the School of Medicine leadership. In 2010, the student scholar positions were advertised to first- and second-year medical students; scholars would be paid a stipend to audit each educational event across that academic year, including lectures, small group discussions, lab sessions, and clinical experiences. Interested students applied with their *curriculum vitae* and a 500-word personal statement describing their interest in the project. The selection criteria were students’ interest in SGBM as described in the personal statement, academic standing, and leadership activities.

**Fig. 1:**
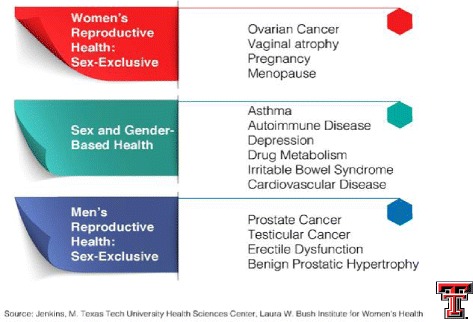
SGBM content versus male or female sex-specific health content

Ten applications were received from a total of 299 students in the MS-1 (*n* = 151) and MS-2 (*n* = 148) classes. The six student scholars selected included two female students and one male student each from both first- and second-year classes. Having three students from each class ensured that at least two students were available to audit each educational event during the year. The student scholars underwent two 1-h training sessions with a faculty member of the SGBM committee who served as the project administrator for the SGBM curriculum audit. During the training, the students were instructed on identifying content about sex or gender differences, rather than material that addressed male or female sex-specific health information (Fig. 1). The students were also given sample lecture handouts and asked to comment on any SGBM content present in the material. In addition, each student scholar selected an SGBM topic of interest and gave a journal club presentation to the committee.

### Real-time student scholar audit of the medical school curriculum for SGBM content

The student scholars were provided with a specific, sharable learning environment within the Blackboard system where they could document in real time all SGBM contents encountered during each learning session. In addition, each team of three student scholars compiled a weekly summary report of all posts utilizing a sharable document file. Since all lectures were recorded and available online for later viewing, the student scholars were allowed the flexibility of reviewing sessions *post facto* but were requested to attend the live session of the lectures as much as possible. After the first 2 weeks of posting, the student scholars again met with the faculty members on the SGBM Committee to provide feedback on the audit process. Based upon the feedback from the student scholars during this meeting, the protocol was adjusted to allow the student scholars to upload lecture slides and notes, assigned reading material, or other PDF materials relevant to each posting.

Initial coding of the qualitative analysis was performed by the students who were extensively trained in categorizing the curricular content into topics or competencies based on Legato’s book. Students coded the topics as “covered,” “not covered,” or “partially covered.” The scholars compiled and reviewed the postings weekly and provided reports to the SGBM Curricular Committee on a monthly basis. As a result of the comprehensive training, there was only a 1–2 % discrepancy rate per each weekly report. Most discrepancies were due to oversight or omission by one or two of the scholars and were resolved by review of the PowerPoint, lecture handouts, or available audio. There were several items on which the scholars could not find agreement, and each was on a topic that was “mentioned in passing” without any actual data or discussion. If the scholars could not reach an agreement as to whether it should be classified as “covered,” the reason for the discrepancy was documented in the report, and the ultimate decision was made by faculty, again based on available PowerPoints, handouts, and audio recordings. Once this information was collected, a separate student scholar not involved in the initial classroom audit verified coding of the text for key terms and identified common themes that were quantified using thematic analysis by a faculty member trained in qualitative analysis.

The real-time student scholar audit was completed for Fall 2010 and Spring 2011 academic sessions. To assure that the students were sampling the baseline “enacted” curriculum [12] and to avoid the observer effect, the lecturers were unaware of the curriculum audit for SGBM content by the student scholars.

### Data analysis

A preliminary and final qualitative analysis was carried out during and immediately following the 2010–2011 academic year. For Summer 2011, an additional student scholar was recruited to review and map the documented TTUHSC SOM SGBM content to the topics covered in Legato’s *Principles of Gender-Specific Medicine*, 2nd ed. [15] (published in 2010), under the guidance of the faculty project administrator. This text was chosen as the basis for the content audit because faculty agreed that it was the most comprehensive and widely accepted source available at the time of the audit. Additionally, its organization allowed for easy formatting of gender-specific medicine competencies using the bolded, non-italicized major section headings in each chapter of the Legato text. These section headings were used in this study to maintain a manageable balance between specificity of content and generality of subject matter which could be more easily matched to the SGBM content in the TTUHSC SOM curriculum. Because *Principles of Gender-Specific Medicine* is a compiled work with more than 100 contributing authors, the style of each chapter varied slightly; thus, some selections in the use of sub-headings were required. The list of SGBM content expected for the first and second years of medical school is available in Additional file 1: Table S1 and Additional file 2: Table S2.

### Annual multi-campus education summit report

In 2011, block or clerkship directors, basic science and clinical faculty, and education deans from each campus were updated on the SGBM Committee’s progress during its regularly scheduled annual education summit. Breakout sessions were utilized to conceptualize, formulate, and consolidate ideas and recommendations that would facilitate the complete integration of SGBM content into the medical school curriculum.

## Results

### Retrospective audit of the existing curriculum for SGBM content

The faculty-generated retrospective review of the syllabus for each block and the review of learning objectives for each lecture did not generate a meaningful catalogue of SGBM content in the curriculum. Most of the content identified was related specifically to women or men and not to sex or gender differences. Evaluating the institutional data in the CurrMIT also yielded little information about the level of SGBM content coverage in the existing curriculum. Some of the entries containing potential SGBM content were in genetics, neurosciences/neuropsychiatry, immunology/infectious diseases, and pharmacology of bone and calcium homeostasis. The lack of appropriate descriptors or systematic approach to tagging SGBM topics resulted only in the identification of “potential hits” with ambiguous status on SGBM content.

The initial analysis of data from the student audit indicated that the existing curriculum included SGBM content focused mainly on anatomical-physiological sex-based differences or gender-based differences in disease prevalence. Analysis revealed gaps in areas such as the approach to treatment of disease and pharmacotherapy. There were very few postings regarding sex- or gender-based differences in diagnosis, prognosis, treatment, and outcomes.

**Fig. 2:**
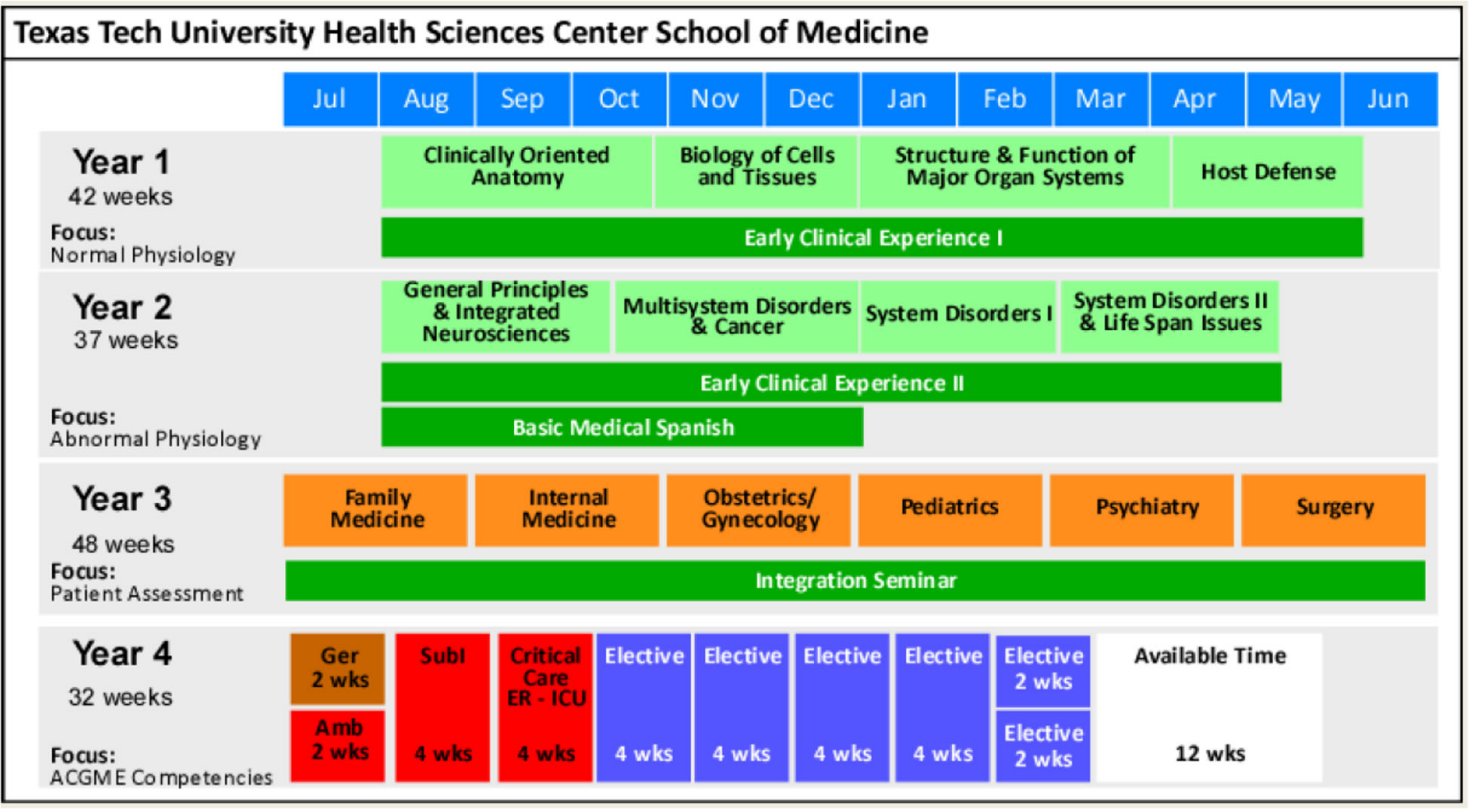
Existing curriculum at the TTUHSC SOM at the time of student scholar audit

**Fig. 3:**
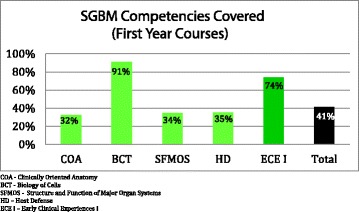
SGBM competencies covered (first-year courses). *COA* clinically oriented anatomy, *BCT* biology of cells, *SFMOS* structure and function of major organ systems, *HD* host defense, *ECE I* early clinical experiences I

**Fig. 4:**
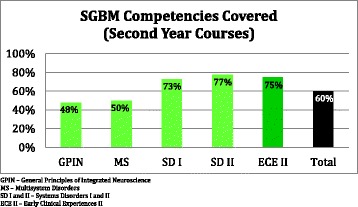
SGBM competencies covered (second-year courses). *GPIN* general principles of integrated neuroscience, *MS* multi-system disorders, *SD I*, system disorder I, *SD II* system disorder II, *ECE II* early clinical experiences II

A more detailed post-audit analysis compared the “enacted” SGBM curriculum (as identified by the student scholars) to an ideal “intended” SGBM curriculum, as defined in the Legato text [15] (Additional file 1: Table S1 and Additional file 2: Table S2). Analysis revealed that, across all five courses, the first-year curriculum at the TTUHSC SOM addressed 41 % of the SGBM topics, while the second-year curriculum addressed 60 % of the topics (Figs. 2, 3, and 4). However, many of those topics (38–70 %), while addressed, were only partially covered.

The real-time audit of the medical school curriculum provided the first detailed mapping of any content in the TTUHSC SOM preclinical curriculum; the audit identified content gaps and facilitated a needs assessment that could inform further integration of SGBM content into the medical school curriculum at all levels.

### Recommendations from the annual Texas Tech medical school education summit

The approach, methodology, and findings of the real-time student audit of the medical school curriculum for SGBM content were presented at the annual TTUHSC SOM summit in 2011. Several major recommendations for integrating SGBM content into the curriculum resulted from summit discussions. The block directors and instructors generally welcomed the opportunity to integrate SGBM content into the curriculum. However, they felt that the greatest barrier to integrating SGBM content into their courses and lectures was the lack of curricular time to insert additional material and of faculty time to research SGBM topics. The faculty expressed concerns regarding the relatively labor-intensive nature of searching for SGBM-specific content in the medical literature since there are no SGBM-specific MeSH terms, so that such searches retrieve a high number of SGBM-non-specific “hits.” Facilitated access to SGBM content was deemed to be critical to its successful integration into the medical school curriculum. In addition, summit consensus recommended that the TTUHSC SOM SGBM Committee develop problem-based learning (PBL) cases that could be integrated into existing blocks and clerkships. Lastly, providing resources and assistance for faculty development was also recommended. These resources included adding SGBM to the annual faculty development offerings, developing continuing medical education (CME) programs, grand rounds, and brown bag sessions for faculty members, and providing financial support and recognition for SGBM teaching, travel for conferences, and student research.

## Discussion

As consideration of sex and gender differences in medicine and health care is critical to providing personalized medicine and improving the outcomes for each patient, incorporating SGBM topics should not be viewed as replacing other “essential” contents. Rather, integrated SGBM instruction in medical schools should be considered essential to better preparing future physicians for providing truly personalized medical care. In addition, standardized exams such as the United States Medical Licensing Examination (USMLE) must adequately test for SGBM content knowledge and skills.

For busy medical educators (including course directors who oversee courses often taught by dozens of clinician or basic sciences faculty members), a complete curriculum audit of any longitudinal theme could prove extremely burdensome. Furthermore, even a retrospective review of course syllabi, presentation learning objectives, or curriculum management databases would be hampered by a lack of appropriate descriptors or an inconsistent approach to tagging SGBM or other contents. The same problem would be expected in the Ilios curriculum management system, which the TTUHSC SOM currently utilizes, since the MeSH headings it uses do not adequately identify or distinguish SGBM content.

The student scholars generally did not perceive the real-time auditing and posting as a significant burden. Having three student scholars for each year of the curriculum and providing them with extensive pre-audit training was critical to an effective capture of SGBM content present in the existing curriculum. As each medical school has different pedagogy and curriculum organizations, adaptation of the student scholar model of auditing may require institution-specific modifications. However, after the curriculum audit for SGBM content within our medical school, other TTUHSC health professions programs have successfully adapted and completed student scholar-driven audits, with minimal modifications or demand on resources.

As a result of the audit of SGBM content, the TTUHSC SOM has taken a number of steps to integrate SGBM into the curriculum and to raise the awareness of sex and gender differences across the TTUHSC. In response to faculty input at the annual education summit, a team of SGBM faculty, student, and library staff developed and validated a freely accessible PubMed-based search tool for identifying sex- and gender-specific medicine and health literature [16]. In development is a slide library of SGBM topics that can be used for basic sciences courses and clinical clerkships; Continuing Professional Development modules on key SGBM clinical issues for physicians, nurses, and other health professionals; and online modules that can be used for a range of learners. Facilitation of access to SGBM-specific literature and instruction material will be critical to successful integration of the SGBM content into the medical school curriculum. A second audit is planned after incorporation of the SGBM curricular tools under development. Hopefully, it will be facilitated by better curriculum management and mapping tools, but a student scholar model would not be out of place.

## Conclusions

Overall, the real-time cataloguing of enacted SGBM curriculum by student scholars was successful in identifying SGBM content not otherwise captured in a review of CurrMIT, course syllabi, or lecture learning objectives. The challenges in retrospectively reviewing the course syllabi, learning objectives, and CurrMIT data were direct consequences of a lack of appropriate descriptors or systematic approach to tagging SGBM content. Therefore, a systematic approach to identifying and documenting SGBM content into the curriculum databases will be critical to facilitating future efforts to assess SGBM content within the curriculum.

The data cataloged by the student scholars allowed for a better understanding of the gaps in SGBM content in the existing medical school curriculum and fostered the development of new instructional methods and resources to correct those deficits.

## Additional files

Additional file 1: Comparative Analysis of MS-1 Student Scholar Audit of SGBM Content in Texas Tech Medical School Curriculum to Key SGBM Topics Outlined in the Principles of Gender Specific Medicine, 2nd Ed. (PDF 121 kb)
Additional file 2: Comparative Analysis of MS-2 Student Scholar Audit of SGBM Content in Texas Tech Medical School Curriculum to Key SGBM Topics Outlined in the Principles of Gender Specific Medicine, 2nd Ed. (PDF 137 kb)
