# Supplementary material for: Auditing sex- and gender-based medicine (SGBM) content in medical school curriculum: a student scholar model
Source: Biol Sex Differ. 2016 Oct 14;7(Suppl 1):40. doi: 10.1186/s13293-016-0102-x (PMC5073909; doi:10.1186/s13293-016-0102-x)
Supplement: Additional file 1: — Comparative Analysis of MS-1 Student Scholar Audit of SGBM Content in Texas Tech Medical School Curriculum to Key SGBM Topics Outlined in the Principles of Gender Specific Medicine, 2nd Ed. (PDF 121 kb) [file 13293_2016_102_MOESM1_ESM.pdf]

Supplemental Table 1. Comparative Analysis of MS-1 Student Scholar Audit of SGBM Content in Texas Tech Medical School Curriculum to Key SGBM Topics Outlined in the Principles of Gender Specific Medici

Key Topics	Covered	Course	Comments
**(1) Effects of Gender in Neonatal Medicine**			
Sex Ratio During Fetal Life	Partially	ECE I (Newborn Clinic), COA (Embryo), SFMOSS	Sex ratio at birth, but not conception, was covered; Predominance of male fetal loss not covered; Predominance of males in premature birth not covered
Excess Male Neonatal Mortality	No	Host Defense	
Postnatal Effects of Changes in the Intra-Uterine Environment	Partially	SFMOS (Repro)	(Sec. 1, p. 8 of P of G-S M)—could be included in Physiology (acidemia, Leydig cells, hypoxia, etc.)
Congenital Anomalies and Gender	Partially	BCT (Genetics)	
Gender and Diseases of Premature Birth	Partially	ECE 1 (Newborn Clinic)	(Sec. 1, p. 10 of P of G-S M)—highlights the Apgar score (covered), Cerebral blood flow, RDS, Septiicemia, Neurobehavior and Pain Perception
Asthma and Gender in Children	No	SFMOS (Respiratory)	
Prognosis for LBW Infants by Gender	No	ECE 1 (Newborn/Peds Clinics)	
**(2) Sexual Development, Growth, and Puberty in Children**			
Normal Sexual Development	Yes	COA (Embryo); SFMOS (Endocrine)	
Disorders of Sexual Development	Yes	COA (Embryo); SFMOS (Endocrine)	
Normal Growth Patterns in Boys and Girls	Yes	SFMOS (Endocrine)	
Abnormal Growth Patterns in Boys and Girls	Partially	BCT (Genetics); SFMOS (Endocrine)	(sec. 1, p. 25 in P of G-B M) External causes of short stature (i.e. psychosocial dwarfism predominantly affecting boys) were not covered
Normal Puberty	Yes	SFMOS (Endo/Repro)	
Variants of Pubertal Development	Yes	SFMOS (Repro)	
Abnormal Puberty	Yes	BCT; SFMOS (Endo/Repro)	
**(3) Gender Differences in Pediatric Pulmonary Disease**			
Developmental Respiratory Physiology	Yes	COA (embryo); SFMOS (respiratory)	
Differences Between Males and Females in Surfactant Production	No	COA (embryo); SFMOS (respiratory)	(sec.1, p.36 in P of G-B M) Greater surfactant protein production in females by 26 weeks, early maturation of female lung, and greater likelihood of males developing transient tachypnea or RDS ("female advantage with respect to RDS in premature infants") not covered
The Role of Sex Hormones on Lung Physiology and the Development of Respiratory Distress Syndrome (RDS)	Yes	SFMOS (Respiratory)	
**(4) Gender-Specific Aspects of Pediatric Hematology and Oncology**			
Normal Hematopoiesis	Yes	BCT (Histology); SFMOS	Differences in male and female hematocrit and hemoglobin were covered
Iron Deficiency Anemia	Yes	BCT (Biochemistry); SFMOS	Most common in menstruating girls
G6PDH Deficiency	Partially	BCT (Genetics/Biochemistry)	The deficiency was covered in the biochemistry unit with respect to clinical manifestations and diagnosis, but the fact that this is a sex-linked disorder primarily affecting males was only briefly mentioned in biochem, not focused on in genetics.
Sickle Cell Disease	Partially	BCT (Genetics)	Median age of death in men is 42 compared to 48 yrs for women. The is a greater rate of pain attacks in males than females. Females have slightly greater fetal Hb which may be protective. Other hormonal differences are noted. (Sec. 1, pp. 52-53 in P of G-B M)
Chronic Granulomatous Disease	No	BCT (Genetics); Host Defense (Immunology)	X-linked disorder affecting mainly boys (not covered). There is also an autosomal recessive form of the disease. Clinical manifestations and pathology of disease were mentioned in Host Defense. (Sec. 1, p. 53 in P of G-B M)
Coagulation Defects	Yes	BCT (Genetics)	The X-linked recessive disorder Classic Hemophilia was covered including the predominance of males in the affected population. (Sec. 1, p. 53 in P of G-B M)
**(5) Gender Differences in Neurological Conditions of Children**			
General Anatomy	Yes	COA	Size differences—new born females and adult females have smaller head circumference on average than the respective aged men; men have more neurons in cerebral cortex, women have more neuropils, etc. (Sec. 1, pp.62-63 in P of G-B M)
DMD	Yes	BCT (Genetics)	X-linked recessive disorder affecting mostly males; Females are mostly at risk of birthing an affected son. (Sec. 1, p. 64 in P of G-B M)
Fragile X Syndrome	Yes	BCT (Genetics)	Mutations on X chromosome, leads to more retardation in males than females. (Sec. 1, p. 64 in P of G-B M)
**(6) Gender and Sports: Past, Present, and Future**			
-Size	Yes	COA; SFMOS	
-Composition	Yes	SFMOS (Cellular)	
-Cardiorespiratory System	Partially	SFMOS (Cardio and Respiratory)	
Male Athlete	No	ECE I	Problems with ergogenic aids. (Sec. 1, p. 70 in P of G-B M)
**(7) Gender Differences in the Functional Organization of the Brain**			
Regional Volumes	No	COA	Hippocampus is larger in women when adjusted for brain size; females tend to generate dendritic spines with more branching compared to males. (Sec. 2, p. 80-81 in P of G-B M)
Cerebral Blood Flow	No	COA	Basal CBF is higher in women. Men activate the right hemisphere for spatial tasks whereas women activate left hemisphere for easy spatial problems and both hemispheres for hard problems. Better verbal memory in women is associated with higher mid-temporal resting CBF. (Sec. 2, p. 81-82 in P of G-B M)
Cerebral Metabolism	No	COA; BCT (Biochem)	There are no gender differences in overall glucose metabolism, however, Sec. 2, p. 82 in P of G-B M explains some regional gender differences in the metabolism of glucose.
**(8) Sexual Differentiation of Brain Structure and Function**			
**(9) The Sexed and Gendered Brain**			
**(10) Age and Gender-Specifiic Patterns of Neurologic Illness**			
**(11) Gender Differences in Stroke**			
Atrial Fibrillation	No	SFMOS (Cardiovascular)	Women appear to have a higher risk of stroke resulting from atrial fibrillation. (p. 130)
Gender Differences in Extracranial and Intracranial Arterial Disease	No	SFMOS (cardio)	Carotid stenosis is more prevalent in men than in women. Men have the maximum surgical benefit; women have 50% higher rates of perioperative stroke and death. In an autopsy series of patients with fatal stroke, men had higher rates of intracranial stenosis than did women. (p. 131)
Inflammation and Stroke	No	SFMOS (Cardio)	Women may be more susceptible to effects from chronic inflammation. Elevated CRP levels are associated with the presence of the metabolic syndrome in women, so this could be a potential mechanism by which inflammation leads to increased atherosclerosis. Chronic inflammation in women could mitigate the protective effect of endogenous estrogens on insulin resistance. (p. 131)

Gender Differences in Stroke symptoms and Recovery from Stroke	No	SFMOS (Cardio)	Women are more likely to have cardioembolic strokes and men are more likely to have lacunar strokes. Women have more disability as a consequence of stroke than men do (may be a partial reflection of their advanced age, as compared to men, when they typically have strokes. (p. 132)
Gender Differences in Mechanism of Stroke	No	SFMOS (Cardio/Repro)	Estrogen acts as a neuroprotectant as demonstrated by the lower rates of stroke in premenopausal women, and the rapid rise in stroke after menopause, as estrogen levels decline. Heritability of stroke may differ in men and women. In one study, women were more likely than men to have a parental history of stroke (primarily maternal).
**(12) Gender Differences in Disorders that Present to Psychiatry**			
**(13) Hormone Replacement Therapy and Cognitive Function**			
**(14) Gender and the Heart: Sex-Specific Differences in the Normal Myocardial Anatomy and Physiology**			
The Myocardium	No	COA	In animal models, testosterone seems to be responsible for the greater LV chamber thickness and wall dimension in males. (p. 152)
The Myofiber	No	COA	It is currently not known whether male myocytes are larger than female myocytes in the normal heart. (p. 152)
Myocardial Hypertrophy	No	SFMOS (Cardio)	(pp. 155-156)
Electrophysiology and Gender	Partially	ECE I (Vitals; SFMOS (Cardio)	Covered: Women have a faster resting heart rate than men.
			Not covered: Hormones may impact the duration of the QT interval (QT interval is shorter in the luteal than in the follicular phase of the menstrual cycle). (pp. 157-158)
**(15) Gender-Specific Aspects of Selected Coronary Heart Disease (CHD) Risk Factors: A Summary of the Epidemiologic Evidence**			
Cigarette Smoking	Partially	ECE I (Lifestyle Habits); SFMOS (Resp)	Covered: high risk of CHD with smoking and benefit of quitting smoking at any age
			Not Covered: smoking rates by gender (women 18% & men 24%) (pp. 162-163)
High Blood Pressure	Partially	ECE I (vitals)	Not Covered: the age-related increase in hypertension, esp. in women
Dyslipidemia	Partially	ECE I; BCT(biochemistry)	Not Covered: serum total cholesterol increases with increasing age; in men, the increase plateaus by age 50, but in women, the increase continues sharply until age 60-65. (pp. 163-164)
Obesity	Yes	ECE I	BMI rates by gender and waste circumference measurements covered. (pp. 164-165)
Physical Activity	Partially	ECE I (lifestyle)	Cardiovascular benefits of moderate-intensity exercise have been observed in men, although they are somewhat weaker than they are in women. (pp. 165-166)
TIID	Partially	SFMOS (Endocrine)	Gender difference in risk of CHD not covered: TIID increases women's risk of developing or dying from CHD up to 7X, whereas it increases men's rise up to 3X. (p. 166)
Aspirin	No	SFMOS (Endocrine)	Women > 65 years old are more likely to receive a net benefit from preventive low-dose aspirin therapy. (p. 167)
Alcohol	No	SFMOS	Women are more susceptible to alcoholic liver disease and to ethanol-induced cardiomyopathy than are men. (p. 168)
**(16) Dyslipidemia Management in Women and Men: Exploring Potential Gender Differences**			
Hormonal Effects on CVD Risk	No	SFMOS (Endo/Repro)	Cited previously (p. 179)
**(17) Gender Differences in the Role of Stress and Emotion in Cardiovascular Function and Disease**			
**(18) The Role of Sex and Gender in Cardiothoracic Surgery**			
Gender and Biology	No	COA; SFMOS (Cardio histology)	
Gender and Risk Factors	No	COA (Clinical Correlate); SFMOS (Cardio)	
Gender and Surgical Outcomes	No	COA (Clinical Correlate); SFMOS (Cardio)	
Gender and Recovery after CABG	No	COA (Clinical Correlate); SFMOS (Cardio)	
Gender and Valve Surgery	No	COA (Clinical Correlate); SFMOS (Cardio)	
Gender and Off-Pump Surgery	No	COA (Clinical Correlate); SFMOS (Cardio)	
**(19) Gender Differences in Asthma**			
Puberty and Asthma	No	ECEI (Peds Clinic); SFMOS (Resp/Clinical)	
Menstruation, Menopause, Hormone Replacement Therapy (HRT) and Asthma	No	SFMOS (Respiratory)	
Animal Models	No	SFMOS (Respiratory)	
**(20) Gender Isssues in Venous Thromboembolism**			
**(21) Sleep in Women: Gender Differences in Health and Disease**			
Sleep Disordered Breathing	Partially	SFMOS (Respiratory)	Typical Obstructive Sleep Apnea (OSA) symptoms were covered, but not mentioned was how women with OSA are often undiagnosed because of a varying clinical presentation. (pp. 246-247)
**(22) Are Women More Susceptible to Chronic Obstructive Pulmonary Disease?**			
Evolving Epidemiology	No	SFMOS (Respiratory)	Prior to the 1960s, most tobacco smokers were men, and additionally men were exposed to many more occupational respiratory irritants than women. This led to a greater preponderance of COPD in men. Now, women in N. America are as likely to smoke as men. Women are projected to require hospitalization for COPD 2X more than men by 2015. (pp. 252-3)
Diagnostic Errors	No	SFMOS (Respiratory)	For many reasons, diagnosis of COPD is delayed which has resulted in an uncertainty in the reported prevalence rates of COPD. (pp. 253-4)
Clinical Manifestations	No	SFMOS (Respiratory)	Women appear to be more greatly impaired than men with comparable disease severity. Health-related quality of life tends to be lower in women and they are more likely to suffer depression. (p. 255)
Management	No	ECE I (Chest Exam and Common Disorders); SFMOS (Respiratory)	There are subtle differences in the responses to therapy between men and women. For instance, women tend to be less successful in smoking cessation efforts than men. (p. 255)
**(23) The Gender-Specific Aspects of Lung Cancer**			
**(24) Gender-Specific Considerations in Pulmonary Hypertension**			

**(25) Sex and Gender Differences in Pulmonary Manifestations of Autoimmune Disease**			
Sex Hormones and Autoimmune Disease	No	Host Defense (Immuno)	Women produce more auto-antibodies than men and have a milder inflammatory response. Estrogens can be proinflammatory or anti-inflammatory and can cause a bimodal effect on cytokines and cells—they can stimulate antibody production by B cells. (277-8)
Rheumatoid Arthritis	No	Host Defense (Immuno)	RA affects more women than men, but RA-related pulmonary complications occur more often in men (3:1). (p. 278)
**(26) Benign Metastasizing Leiomyoma and Lymphangioleiomyomatosis: Lung Disease of Women**			
**(27) Gender Differences in Susceptibility, Outcomes, and Pathophysiology of Sepsis**			
Epidemiology	No	Host Defense (Immuno)	Sepsis appears to be more common in men than women. Men are more likely to develop sepsis after surgery.
Sex Hormones and the Immune Response to Stress	No	Host Defense (Immuno)	Estrogen in animal models plays a protective role by stimulating humoral and cell-mediated immunity. (293-294)
Gender Differences in Hemodynamic Response to Stress	No	Host Defense (Immuno)	Estrogen and relaxin may reduce pulmonary artery pressures and improve hemodynamics. Testosterone, on the other hand, acts as an immune suppressant. (p. 295)
Gender Differences and Gene in Polymorphisms	No	Host Defense (Immuno)	One gene involved in mediating sepsis X-linked which leads to an increased susceptibility for males. (pp. 296-297)
Health Care Access and Delivery	No	Host Defense (Immuno)	Women were found to seek preventive care and chronic care, but not acute care, more often then men. (p. 297)
**(28) Inflammatory Bowel Disease in Women**			
Menstrual Abnormalities	No	COA; SFMOS (GI)	The majority of women with IBD experience menstrual abnormalities. Many adolescent girls with IBD will experience menarche later than healthy girls (due to malnutrition, disease, etc.). Iron deficiency is a common complication of IBD. (p. 305)
Rectovaginal Fistulas	Yes	COA; SFMOS (GI)	Covered in COA (p. 305)
Osteoporosis	No	COA; SFMOS (GI)	Screening for osteoporosis should begin within 6 mo. of a diagnosis of IBD. Decreased bone mineral density in IBD is likely related to malnutrition and systemic inflammation, and probably corticosteroid use.
Self-Image	No	COA; SFMOS (GI)	IBD patients often experience physical symptoms that are not socially acceptable which causes psychosocial complications. (p. 313)
**(29) Disorders of Defecation in Women**			
Prevalence and Etiology of Constipation	No	COA (Clinical Correlation); SFMOS (GI)	Constipation is one of the most common digestive complaints.
Evaluation of Constipation	No	COA (Clinical Correlation)	
Treatment of Constipation	No	COA (Clinical Correlation)	Physical exam in up to 81% of elderly women will demonstrate a rectocele. (p. 320)
Prevalence and Etiology of Fecal Incontinence	No	COA (Clinical Correlation)	The most common cause of fecal incontinence is anorectal trauma related to childbirth. An anatomical defect may occur in up to 32% of women following parturition regardless of visible damage to the perineum.
Evaluation of/ Treatment of Fecal Incontinence	No	COA	The sphincter anatomy is different when evaluating females v. males. In females, there is loss of the normal external sphincter complex in the upper anal canal. This is not a loss secondary to childbirth but is present because the rectovaginal septum creates the upper canal. (p. 322)
**(30) Idiopathic Gastroparesis: Gender Aspects**			
Normal Gastric Emptying	Yes	SFMOS (GI)	Normal gastric emptying was covered. (p. 326)
Gender Aspects of Gastric Motility	No	SFMOS (GI-Clinical)	Gastric emptying in premenopausal females is delayed compared to that in males. Gastric emptying may be slower during the luteal phase (days 18-20) when estrogen and progesterone levels are elevated as compared to the follicular phase (days 8-10). Post-menopausal women on HRT have slower gastric emptying of solids than men. Feale reproductive hormones likely have inhibitory effects on gastric motility. Nausea of pregnancy occurs when estradiol and progesterone are elevated and is associated with gastric dysrhythmias. (p 326)
Gastroparesis	Yes	SFMOS (GI)	Basics of gastroparesis were covered. (p. 327)
Gastric Motility Abnormalities in Gastroparesis	No	SFMOS (GI-clinical)	Most patients with gastroparesis are women; typically young or middle aged. (p. 328)
**(31) Liver Diseases in Women**			
Pregnancy and Liver Disease	No	SFMOS	OHSS is a liver disease unique to pregnancy; it is a potentially fatal iatrogenic complication associated with ovulation –induction therapy. Hepatitis E can be a major epidemic in pregnant women. Pregnancy is an independent risk factor for cholelithiasis because increased progesterone decreases gallbladder motility and emptying. Childbearing after liver transplantation is uncommon and a high-risk pregnancy. (p. 331)
Autoimmune Liver Diseases	No	Host Defense (immune)	Autoimmune hepatitis and primary biliary cirrhosis are autoimmune diseases predominantly affect the female population. (p. 337)
Liver Transplantation and Surgery	No	Host Defense (Immuno)	Livers from female donors were found in many studies to yield poorer graft survival rates than livers from male donors. (p. 339)
**(32) Gender Differences in Irritable Bowel Syndrome**			
**(33) Contraception**			
Contraceptive Counseling	Yes	ECE 1; SFMOS (Repro)	In adolescents and adults continuation rates may be improved by reviewing how contraceptives work and their expected side-effects, by giving oral and written instructions, and especially by providing samples at the initial visit and ample refills. (p. 357)
Contraceptive Methods	No	ECE 1; SFMOS (Repro-Clinical Correlate)	The various contraceptive methods were discussed in pp. 357-363.
**(34) Infertility: The Male**			
Treatment	Partially	SFMOS (Repro)	Covered: ICSI; Not Covered: Transurethral resection of the ejaculatory ducts (TURED), HRT with recombinant FSH. (p. 375)
**(35) Infertility and In Vitro Fertilization**			
Treatment for Infertility	No	COA (Repro-Clinical); SFMOS	Clomiphene citrate or injectable gonadotropins can be considered for treatment of ovulation dysfunction. Tubal obstruction can be laparoscopically corrected. IUI and IVF cervical problems, tubal problems, endometriosis, ovulatory disorders, and more. (p. 383)
**(36) Female Sexual Health**			
Anatomy and Physiology	Yes	COA (Pelvis and Perineum); SFMOS (Reproductive)	The anatomy and physiology of the clitoris, vulva, vagina, and uterus were detailed. Not a great deal is known about the exact nature of the afferent innervation of the female genital. (pp. 400-402)
Clinical Practice	No	COA (Pelvis and Perineum); SFMOS (Reproductive)	Risk factors for female sexual dysfunction are hypertension, smoking, hyperlipidemia, and endothelial dysfunction. (p. 402)

Common Sexual Problems	No	COA (Pelvis and Perineum); SFMOS (Reproductive)	Hypoactive Sexual Desire Disorder is one of the most common sexual problems among women. Other disorders are Female Sexual Arousal Disorder, Female Orgasmic Disorder, Dyspareunia, and Vaginismus. (pp. 403-404)
Treatment	Partially	COA (Repro-Clinical); SFMOS	Covered: Kegel exercises help to strengthen the pubococcygeus muscle supporting the pelvic floor; Not covered: Various pharmacological agents are also used in treating FSDs. (pp. 405-407)
Physiology	Yes	COA (Pelvis and Perineum); SFMOS (Reproductive)	
**(37) Male Sexual Dysfunction**			
Definition and Pathophysiology of Erectile Dysfunction	Partially	COA (Pelvis and Perineum); SFMOS (Reproductive)	There are 3 classifications of ED: psychogenic, organic, and mixed.
Epidemiology	No	COA (Pelvis and Perineum); SFMOS (Reproductive)	Prevalence of ED in men 40-70 was found in the MMA study to be 52%. (p. 409)
Risk Factors	No	SFMOS (Reproductive)	DM, Heart disease, hypertension, dyslipidemia, lower urinary tract symptoms, and cigarette smoking. (p. 409)
Evaluation	No	SFMOS (Repro Clinical)	Risk factors are assessed and questionnaires and physical exams and tests are conducted. (p. 410)
Special Tests	No	SFMOS (Repro Clinical)	Nocturnal erections occur in every healthy man of all ages. 80% occur in REM sleep. the Nocturnal Penile Tumescence Test can be used for medicolegal conditions. (p. 410)
Non-surgical Therapy: PDE5I, Urethral Suppositories, ICI, VED	Partially	SFMOS (Repro Clinical)	Covered: Mechanism of PDE-5 Inhibitors.<br>Not covered: Urethral Suppostitories, vaccum erection devise. (pp. 410-411)
Surgical Therapy	Partially	COA (Clinical)	ED in young men with isolated stenosis or occlusion of penile arteries as a result of pelvic/perineal trauma is amenable to surgical repair. (p. 411)
**(38) Pelvic Pain: Urogenital Female Disorders**			
**(39) Lower Urogenital Tract Dysfunction in Men and Women**			
Lower Urinary Tract Dysfunction: Definitions, Symtoms, and Classification	Yes	ECE I; COA (Pelvis & Perineum)	Women have a shorter urethra making them more prone to UTIs and incontinence. The male urethra is much larger and has 4 parts. Due to it's length, the male urethra is more prone to traumatic injury and stricture formation. (pp. 421-423)
Conditions Causing Lower Urinary Tract Symptoms in Men, Women, and both Sexes according to Symptoms	Partially	ECE I; COA (Pelvis & Perineum)	(pp. 423 – 425)
Assessment of Lower Urinary Tract Dysfunction in men and women	Partially	ECE 1	Uroflowmetry is very useful in men to help quantify obstruction whereas in women obstruction is less common. In women, because of the close proximity between the bladder, urethra, uterus, and vagina, incontinence an dpelvic organ prolapsed are interrelated. (pp. 425-427)
Age-Related Changes in the Lower Urinary Tract	Partially	SFMOS (Repro)	Covered: The postmenopausal decrease in estrogen plays a part in many age-associated vaginal changes. Estrogen is trophic for much of the lower urinary track in women, with estrogen receptors found in the vagina, vestibule, distal urethra, bladder trigone, pelvic muscles, and ligamentum rotundum. Following menopause, the superficial and intermediate layers of the vaginal epithelium thin and may disappear.<br>Not covered: There are 2 major conditions in the aging bladder: overactive detrusor urinae muscle and decreased detrusor contraction strength. (p. 434)
**(40) Aging and the Lower Urogenital System**			
Urinary Tract Infections	Partially	COA (P & P)	Urinary Tract Infections are extremely common in older persons and more prevalent in younger women compared to younger men (40:1). (p. 436)
Urinary Incontinence (UI) in Older Persons	No	COA; Physio (repro)	UI increases with age. There seems to be an ethnic variation in the prevalence of UI in women, but not men (more white women show effects of UI than black women). (p. 437)
**(41) Menopause**			
Physiology	Yes	SFMOS (Repro)	(p. 450)
Evaluation of Women	No	SFMOS (Repro Clinical)	Women should be counseled on their own expectations of menopause, baseline studies including mammography and bone density should also be done. (p. 451)
Role of Hormone Therapy	Partially	SFMOS (Repro Clinical)	Covered: HRT is a treatment option for some menopausal women. HRT can be administrated orally, transdermally, or locally. Before commencint HRT< a woman's benefit:risk ratio should be considered.<br>Not covered: HRT may reduce total mortality when initiated in women under age 60 years. (p. 453)
**(42) The Differences between Male and Female Breast Cancer**			
Anatomy and Development	Yes	ECE I	Rate of breast cancer is much higher in women in part because of the greater development of breast tissue in women. Male and female breasts are similar at birth, and they diverge at the time of puberty. In males development ceases. (p. 459)
Risk Factors	Partially	BCT (Genetics)	Covered: BRCA susceptibility genes are linked to breast cancer<br>Not Covered: early menarche, shorter cycle length, nulliparity, or low parity, and late menopause are several reproductive variables that increase a woman's risk for developing breast cancer. (p. 460)
**(43) Difference in Germ Cell Tumors of the Reproductive Tract in Men and Women**			
Origin of Germ Cells	Yes	COA (Embryo)	Mullerian duct forms the female internal reproductive organs, and the Wolffian duct gives rise to het male reproductive organs
Gender Differences in the Epidemiology of Germ Cell Tumors	No	COA (Embryo-Clinical)	Ovarian germ cell tumors account for 25% of all pediatric GCTs whereas testicular tumors comprise 10% of pediatric GCTs. GCTs are rare in adult females, however they are the most common solid tumors in men between the ages of 15 and 34 years. Female GCTs are more prevalent in blacks and Asians whereas male GCTs are more prevalent in whites.(p. 474)
Biology and Risk Factors for Germ Cell Tumors: Are they Gender-specific?	No	BCT (Genetics)	Virtually 100% of male GCTs show the same abnormal increased number of copies of the 12p chromosome. This chromosome marker is present in the first recognizable stage of GCT development, carcinoma in situ, suggesting that this abnormality may be the earliest genetic change in the development of GCTs. (pp. 475-476)
**(44) Gender Differences in Hereditary Cancer Syndromes: Risks, Management, and Testing for Inherited Predisposition to cancer**			
Cancer Genetics: Overview	Yes	BCT (Genetics)	Regardless of whether an individual manifests disease, individuals can still pass on this mutation to the next generation. This has important implications when taking a family history to assess risk of cancer because susceptibility is equally likely to be inherited through the paternal as the maternal side. (p. 482)
Breast Cancer	Partially	BCT (Genetics)	Not Covered: Peutz-Jeghers Syndrome—the lifetime risk of all cancer in carriers is 18X higher in women and 6.2X higher in men. (pp. 483-485)
Endometrial and Gastrointestinal Cancer	Partially	BCT (Genetics)	Men with Hereditary Nonpolyposis Colon Cancer (HNPCC) mutations are most likely to develop colon cancer, whereas women with HNPCC are most likely to develop endometrial cancer. (pp. 485-487)
**(45) Gender Differences in Emerging Infectious Diseases**			

Emerging Viral Infections	No	Host Defense (Micro)	Nipah virus covered, but not covered was the 4.5:1 male:female ration in the first outbreak. In pregnant women with Chikungunya infection, viremic women were more likely to transmit the virus to their neonate, and cesarean section did not protect against transmission. Gender differences with Hantavirus, Monkeypox, and West Nile Virus were also covered. (pp. 498-505)
Emerging Bacterial Infections	Partially	Host Defense (Micro)	More than 70% of Bartonella endocarditis cases have occurred in men. No observable male:female differences in EHEC. (p. 505)
Emerging Prion Infection	No	Host Defense (Micro)	No preponderance in either sex has been reported for CJD, but one prion disease, kuru, is known to be more prevalent in women. (p. 511)
**(46) Sexually Transmitted Infections in Men and Women**			
Gender Differences in Behavioral Susceptibility	Yes	Host Defense	Women are generally considered more responsible for pregnancy prevention. (p. 516)
Gender Differences in Biological Susceptibility	No	Host Defense	Male circumcision status is one of the most important characteristics associated with variable risk of STIs. The foreskin contains large numbers of dendritic cells which are a target for HIV. It may also act as a reservoir for sexually transmitted pathogens that could lead to urethritis. (p. 517)
Sexually Transmitted Bacterial Infections	Yes	Host Defense (Micro)	N. gonorrhoeae, C. trachomatis, Syphillis and chancroid were all covered. (pp. 520-524)
Sexually Transmitted Viral Infections other than HIV	Yes	Host Defense (Micro)	Women consistently have a higher rate of HSV-2 seropositivity than men (perhaps due to the higher rate of male-to-female transmission compared to female-to-male transmission. (p. 524-526)
Other Organisms and Syndromes	Partially	Host Defense (Micro)	Covered: N. gonorrhoeae, C. trachomatis, M. hominis, U. urealyticum, Bacteroides, E. coli organisms' involvmenint in PID. not covered: M. genitalium. >100,000 women annually become infertile as a result of PID. (pp. 527-528)
**(47) Infections in Pregnancy**			
Mechanisms of Increased Susceptibility to Infection in Pregnancy	No	Host Defense	Changes in the immune response to infection are an inadvertent byproduct of the alterations in maternal immune function that are essential in order to initiate and sustain pregnancy. There is a 'type 1' to 'type 2' shift in maternal immune function during pregnancy. The increased steroid hormone levels in pregnancy lead to a decrease in the number of circulating lymphocytes. (pp. 531-536)
Infections for which Pregnancy Alters Maternal Susceptibility or Course	Partially	Host Defense	UTIs, pneumonia, puerperal infections, , Hep E, malaria, and Shistosomiasis were covered. (pp. 536-544)
Infections in which Fetal Transmission or other Fetal Morbidity is the Primary Concern	Partially	Host Defense	T. gondii actively invades cells and can cross biological barriers including the placenta and blood brain barrier. Congenital toxoplasmosis causes a disseminated infection in the fetus, and T. gondii DNA can be detected in amniotic fluid, blood, cerebrospinal fluid, and other fetal tissues. (p. 544) CMV infection during the 3rd trimester has the highest rate of congenital transmission. (p. 545) HSV infection is more severe in pregnancy. Maternal antibody is protective. Parvovirus B19 causes the "fifth disease" in childhood. Fetal infections can lead to severe fetal anemia, fetal hydrops, and fetal loss in 3rd trimester. (p. 547) VZV in a fetus is highly fatal because the fetus is not yet able to mount a CMI response. (p. 548) (several other infections are also covered (pp. 549-554)
Emerging Infections	Partially	Host Defense	Dengue Hemhorragic fever is more likely to follow a primary dengue infection in pregnant than in non pregnant women. (p. 555) It is indconclusive whether a WNV infection in pregnant women in the US relates to developmental abnormalities. For pregnant women with Lassa fever v, the greatest risk of infant death is in the 3rd trimester. We did not cover lymphocytic Choriomeningitis Virus. (p. 555)
Potential Agents of Bioterrorism	Yes	Host Defense	Smallpox is a concern for bioterrorism, and historically it has much higher mortality rates in pregnant women. (p. 556)
Vaccination	Yes	Host Defense	Attenuated live vaccines should be avoided since there is risk of dissemination to the fetus. There are no known risks to vaccination of pregnant women with bacterial vaccines or toxoids or with inactivated virus vaccine. (p. 556)
**(48) Adult Immunization in Women and Men**			
Human Papillomavirus G	Yes	Host Defense	>50% of sexually active people over 50 years old acquire HPV at some point in their lives. Gardisil is currently recommended for girls 9-26, but it is starting to be recommended for males as well. (pp. 563-565)
Zoster Immunization	Yes	Host Defense	Confirmed zoster cases were 11% higher among US women in a cohort of immunocompetent persons 60 years and older. (p. 566)
Influenza Vaccine	Yes	Host Defense	Influenza results in more significant mortality in women in the 2nd and 3rd trimester of pregnancy. Vaccination is recommended (killed vaccine only) for pregnant women. (p. 568)
Hepatitis B Vaccine (HBV)	Yes	Host Defense	Men seem to be at a higher risk of developing chronic hepatitis B virus infection after exposure as compared to their female counterparts, and also had higher rates of development of hepatocellular carcinoma. (p. 568)
Hepatitis A Vaccine (HAV)	Yes	Host Defense	There is a particular risk for HAV infection for men who have sex with men. (p. 569)
Pneumococcal Vaccine	Yes	Host Defense	(p. 570)
Tetanus/Diptheria/Pertussis	Yes	Host Defense	One of the WHO's biggest concerns is with maternal and neonatal tetanus. (p. 572)
Meningococcal Vaccine	No	Host Defense	(p. 573)
Immunization Issues for Pregnant Women	Yes	Host Defense	Potential risks associated with vaccinating a pregnant woman: inadvertent infection of the unborn fetus, possible teratogenicity of the vaccine, lack of efficacy of the vaccine. (p. 574)
**(49) Gender Differences in Autoimmune Diseases: Immune Mechanisms and Clinical Applications**			
Specific Remarks in Autoimmunity	No	Host Defense (Immunology)	There is altered estrogen metabolism in SLE patients. SLE patients have increased 16-hydroxylation of estrogen. (p. 585)
Specific Autoimmune Diseases with Gender Differences	Partially	Host Defense (Immunology)	Females with Myasthenia gravis have an altered cell population in the thymus. (p. 588)
Future Applications and Concluding Remarks	No	Host Defense (Immunology)	(p. 589)
**(50) Hormones and Cytokines: Gender-Specific Effects**			
Involvement of Sex Hormones and Cytokines in Autoimmune Rheumatic Diseases	Partially	Host Defense (Immunology)	Women are more often affected by autoimmune rheumatic diseases than men. Sex hormones are involved in the immune response, and estrogens enhance humoral immunity whereas androgens and progesterone function as natural immunosupressors. (p. 592)
Peripheral Sex Hormone Metabolism in Autoimmune Diseases	Partially	Host Defense (Immunology)	Aromatase is increases in RA patients and conversion of upstream androgen precursors to 17-B-estradiaol is accelerated. (p. 593)
Sex Hormones and Cytokines	No	Host Defense (Immunology)	Macrophage release of proinflammatory cytokines can be modulated by estrogen particularly by modulation of CD16 expression. (p. 594)
**(51) Prolactin and Autoimmunity**			
Prolactin	No	Host Defense (Immunology)	Estrogen is a potent stimulus for production of pituitary prolactin in rodents, and estrogen stimulates autoimmunity in lupus model rats. The presence of prolactin is necessary in order for estrogen to exert its stimulatory effects on autoimmune disease. (pp. 597-599)
Prolactin and Autoimmune disease	No	Host Defense (immunology)	Hyperprolactinemia can stimulate autoantibodies in individuals without clinical autoimmune disease. (p.599)
Prolactin-lowering Therapy for Autoimmune Disease	No	Host Defense (Immunology)	No gender differences in prolactin-lowering therapy were discussed. (p. 607)

**(52) Sex Hormones and Immune Function**			
Normal Estrogen and Androgen Metabolism	Yes	SFMOS (endocrinology)	pp. 617-620
Use of Hormones to Alter Disease States	No	SFMOS (endocrinology); HD (immunology)	Most studies involving oral contraceptives and SLE or hormone replacement therapy in post—menopausal women with SLE suggest that the disease worsens with these agents. (p. 621)
Sex Hormones, Behavior, and Autoimmune Diseases	No	SFMOS (endocrinology)	(pp. 621-622) Brain development is dependent upon sex steroids. SLE mice may have aberrant neuronal migration leading to dyslexia or left-handedness). There is a greater prevalence of left handedness in the SLE population than in the unaffected population.
**(53) Pregnancy and Autoimmune Rheumatic Disease**			
**(54) Oral Contraceptives and Autoimmune Diseases**			
**(55) Gender-Specific Issues in Organ Transplantation**			
Organ Failure	No	Host Defense (Immunology)	Men with autoimmune hepatitis appear to have a higher relapse rate and younger age of disease onset. Despite this, men have significantly better long-term survivial and outcomes. Oral contraceptives have been associated with several hepatobiliary diseases. (p. 657)
Organ Donation	No	Host Defense (Immunology)	Men are more ambivalent about organ donation. 73% of spousal donations are wife to husband. (p. 660)
Outcome Post-Transplantation	No	Host Defense (Immunology)	Livers from female donors yield significantly poorer results than those from male donors. (p. 661)
**(56) Endogenous Sex Hormones and Risk of Type 2 Diabetes Mellitus in Men and Women**			
**(57) Thyroid Disorders and Pregnancy**			
Hypothyroidism	No	SFMOS (Endocrinology)	Untreated hypothyroidism is known to be associated with an increased risk of infertility and may be associated with increased rates of miscarriage after conception. (p. 696)
Hyperthyroidism	No	SFMOS (Endocrinology)	Gestational transient thyrotoxicosis appears to be precipitated by excessive stimulation is predominantly caused by the secretion of abnormally high levels of normal hCG or the production of variant forms that demonstrate greater affinities for thyroid tissue. (p. 699)
Thyroid nodules and Thyroid Cancer	No	SFMOS (Endocrinology)	Palpable thyroid nodules may become more apparent during pregnancy. (p. 702)
**(58) Sexual Function and Dysfunction in Men and Women**			
**(59) Osteoporosis in Men and Women**			
**(60) Testosterone Replacement Therapy in Men and Women**			
Androgen Physiology	Yes	SFMOS (Endocrinology)	p. 737 Evidence has shown that testosterone may play a role beyond masculinity in both men and women.

ECE I: Early Clinical Experience
COA: Clinical Oriented Anatomy
BCT: Biology of Cells and Tissues
SFMOS: Structure and Function of Major Organ Systems
HD: Host Defense
