# Supplementary material for: Auditing sex- and gender-based medicine (SGBM) content in medical school curriculum: a student scholar model
Source: Biol Sex Differ. 2016 Oct 14;7(Suppl 1):40. doi: 10.1186/s13293-016-0102-x (PMC5073909; doi:10.1186/s13293-016-0102-x)
Supplement: Additional file 2: — Comparative Analysis of MS-2 Student Scholar Audit of SGBM Content in Texas Tech Medical School Curriculum to Key SGBM Topics Outlined in the Principles of Gender Specific Medicine, 2nd Ed. (PDF 137 kb) [file 13293_2016_102_MOESM2_ESM.pdf]

Supplemental Table 2. Comparative Analysis of MS-2 Student Scholar Audit of SGBM Content in Texas Tech Medical School Curriculum to Key SGBM Topics Outlined in the Principles of Gender Specific Medicine, 2nd Ed.

Key Topics	Covered	Course	Comments
**(1) Effects of Gender in Neonatal Medicine**			
Sex Differences in Fetal and Neonatal Growth	Yes	MS	
Excess Male Neonatal Mortality	No	MS	
Early Metabolic Programming	Yes	MS	
Congenital Heart Disease and Outcomes of Cardiac Surgery	No	SD I	
Asthma and Gender in Children	Yes	SD I	
**(2) Sexual Development, Growth, and Puberty in Children**			
Disorders of Sexual Development	Yes	SD II	
Variants of Pubertal Development	Yes	SD II	
Abnormal Puberty	Yes	SD II	
**(3) Gender Differences in Pediatric Pulmonary Disease**			
Developmental Respiratory Physiology	Yes	SD I	
Fetal Breathing and Lung Maturation	Yes	MS	(sec.1, p.36 in *P of G-B M*)—Fetal lung maturation is reflected in fetal mouth movements; Females demonstrate significantly more fetal mouth movements than males at each stage of development indicating differences in lung maturation between females and males in the prenatal period.
The Role of Sex Hormones on Lung Physiology and the Development of Respiratory Distress Syndrome (RDS)	Yes	MS	
Sex Differences in Alveolar and Airway Development	Yes	SD I	(Sec. 1, p.37 in *P of G-B M*) The smaller lung size of girls and fewer respiratory bronchioles in girls were not covered. These sex differences are important risk factors for numerous respiratory diseases in childhood, and they explain differences in prevalence and severity of a variety of pulmonary diseases.
Lung Function in Infancy and Childhood	Yes	MS	(Sec. 1, p.38 in *P of G-B M*) Not covered: Greater forced expiratory flow rates in girls during childhood and early adolescence, Greater lung function in males during late adolescence
Asthma	Yes	SD I	Not covered: Males have higher prevalence of asthma under 6 yrs; Increased morbidity from lower RT disease in boys (partially due to increased resting airway tone in boys); By late adolescence greater prevalence of asthma in females. Boys are 2X more likely to be diagnosed with asthma even when presenting same symptoms as girls. Also smoking, atopic responses, and hormonal influences are mentioned.(Sec. 1, pp.38-40 in *P of G-B M*)
Cystic Fibrosis	Yes	MS, SD I	Median survival age for females is 3-4 years less than for males. There is a gender gap in the age at diagnosis with girls being diagnosed 18 months later when presenting with respiratory symptoms. Colonization of girls with *P. aeruginosa* precedes the colonization in boys. Also mentioned are gender differences in mortality rates for patients based on dietary intake, nutritional status, and fitness, co-morbidity with CF-related diabetis, and sex hormones. (Sec. 1, pp. 40-43 in *P of G-B M*)
Sleep Disorders	No	GPIN	In Respiratory Physiology, obstructive sleep apnea was presented, but the following gender-specific characteristics were not covered: males have a higher prevalence; women with same symptoms are often misdiagnosed with depression. Women may have greater patency of the upper airway during sleep (Sec. 1, pp. 43-46 in *P of G-B M*)
**(4) Gender-Specific Aspects of Pediatric Hematology and Oncology**			
Iron Deficiency Anemia	Yes	MS	Most common in menstruating girls
G6PDH Deficiency	Yes	MS	The deficiency was covered in the biochemistry unit with respect to clinical manifestations and diagnosis, but the fact that this is a sex-linked disorder primarily affecting males was only briefly mentioned in biochem, not focused on in genetics.
Sickle Cell Disease	Yes	MS	Median age of death in men is 42 compared to 48 yrs for women. The is a greater rate of pain attacks in males than females. Females have slightly greater fetal Hb which may be protective. Other hormonal differences are noted. (Sec. 1, pp. 52-53 in *P of G-B M*)
Coagulation Defects	Yes	MS	The X-linked recessive disorder Classic Hemophilia was covered including the predominance of males in the affected population. (Sec. 1, p. 53 in *P of G-B M*)
Childhood Cancer	Yes	MS	More frequent in males than females. Excess mortality in males. Male preponderance of non-Hodgkin's lymphoma, acute lymphoblastic leukemia, acute myeloidleukemia, CNS embryonal and gem cell tumors, hepatoblastoma, hepatocellular carcinoma, rhabdomyosarcoma, ad gonadal germ cell tumors. Thyroid cancer is 5X more frequent in females than males. Malignant melanoma affects 20% more girls than boys. (Sec. 1, pp. 53-58 in *P of G-B M*)
Cancer Risk Factors	Yes	MS	Risk factors such as X-linked immunodeficienceies, cryptorchidism, and more were mentioned. (Sec. 1, pp. 54-55 in *P of G-B M*)
Cancer Survival Outcome	No	MS	Girls have a better prognosis for surviving Acute lymphoblastic leukemia. (Sec. 1, pp. 55-56 in *P of G-B M*)
Acute and Late Effects of Treatment	No	MS	Girls experience a higher incidence of cardiac abnormalities at any cumulative dose of doxorubicin, perhaps due to relatively more body fat resulting in decreased clearance of drugs, prolonging exposure. Many other effects of treatment are mentioned. (Sec. 1, pp. 56-57 in P of G-B M)
**(5) Gender Differences in GPINlogical Conditions of Children**			
ADHD	Yes	GPIN	More prevalent in boys; Girls with ADHD are more likely to have lower IQ scores, anxiety disorders, and other co-morbidities listed in the text. (Sec. 1, p. 63 in *P of G-B M*)
Tourette's Syndrome (TS)	Yes	GPIN	Up to 10X more common in boys. (Sec. 1, p. 63 in *P of G-B M*)
Epilepsy	Yes	GPIN	Higher incidence in males for most types of seizures; Epilepsy is also seen in the X-linked dominant Aicardi and Rett syndromes (occur mostly in females) (Sec. 1, pp. 63-64 in *P of G-B M*)
Pelizaeus-Merzbacher Disease (PMD)	No	MS	Seen predominantly in males, X-linked recessive disorder. (Sec. 1, p. 64-65 in *P of G-B M*)
**(6) Gender and Sports: Past, Present, and Future**			
Physiology	Yes	SD II	
-Body Proportions	Yes	SD II	
-Composition	Yes	SD II	
-Cardiorespiratory System	Yes	SD I	
Female Athlete	Yes	SD II	"Female athlete triad": Disordered eating, amenorrhea, and osteoporosis esp. in gymnastics, ballet, figure skating, and cross-country running. Tend to be deficient in B vitamins. (Sec. 1, p. 69-70 in *P of G-B M*)
Male Athlete	No	SD II	Problems with ergogenic aids. (Sec. 1, p. 70 in *P of G-B M*)
**(7) Gender Differences in the Functional Organization of the Brain**			
Cognitive Processing	Yes	GPIN	Women perform better on some verbal and memory tasks; men excel in spatial tasks—differences attributed to variation in hemispheric specialization of cortical function. (Sec. 2, p. 75-76in *P of G-B M*)
Emotion Processing	Yes	GPIN	Women perform better I emotion recognition tasks; Women are more prone to clinical depression; Memory of emotional material involves mostly right hemisphere of the amygdale in males and lest hemisphere of amygdale in females. (Sec. 2, p. 77-78in *P of G-B M*)
Psychological Stress Response	No	GPIN	Males tend to have the 'fight or flight' response, whereas females tend to have the 'tend-and-befriend' response. (Sec. 2, p. 78in *P of G-B M*)
Whole Brain	No	GPIN	Men have more white matter than women. Women have a greater percentage of GM. (Sec. 2, p. 79 in *P of G-B M*)
Regional Volumes	No	GPIN	Hippocampus is larger in women when adjusted for brain size; females tend to generate dendritic spines with more branching compared to males. (Sec. 2, p. 80-81 in *P of G-B M*)
Cerebral Blood Flow	No	GPIN	Basal CBF is higher in women. Men activate the right hemisphere for spatial tasks whereas women activate left hemisphere for easy spatial problems and both hemispheres for hard problems. Better verbal memory in women is associated with higher mid-temporal resting CBF. (Sec. 2, p. 81-82 in *P of G-B M*)
Cerebral Metabolism	No	GPIN	There are no gender differences in overall glucose metabolism, however, Sec. 2, p. 82 in *P of G-B M* explains some regional gender differences in the metabolism of glucose.
GPINreceptors	No	GPIN	Studies in rats have shown that stress enhances performance and increases the number of dendritic spines in male rats although it diminishes performance and decreases the density of dendritic spines in female rats. (Sec. 2, p. 82 in *P of G-B M*)
Implications for Sex Differences in Functional Brain Organization	No	GPIN	(Sec. 2, p. 82-83 in *P of G-B M*)
**(8) Sexual Differentiation of Brain Structure and Function**			
Volumetric Sex Differences	No	GPIN	Overall larger male brain size, greater number of GPINns contributing to a greater SDN-POA(sexually dimorphic nucleus of the preoptic area). The SDN-PDA is the most famous brain sex difference since it is 5-7X larger in males. The difference is due to estradiol during perinatal period.
Synaptic Patterning Sex Differences	No	GPIN	The male pattern of synapses in the preoptic area and ventromedial nucleus is essential for the normal expression of male sexual behavior in adulthood. (p.90) In rodents, three is a marked male bias in the density of vasopressin innervation of the septum. Vasopressin is strongly associated with the control of affiliative behaviors such as parenting and pair bonding. The denser vasopressin innervation in males is speculated to provide the GPINlogical underpinnings for inducing males to provide parental care of their offspring, an endpoint achieved in females by the hormonal milieu of pregnancy, parturition, and lactation. (p. 91)
A Critical Role for Naturally Occurring Cell Death	Yes	GPIN	Sex differences in number of GPINns are established when males and females begin with the same number of GPINns but that differential hormonal exposure results in sex differences in cell death. (p.91)
An Emerging Role for Cell Genesis and/or Cell Differentiation	No	GPIN	(p. 92)
Importance of Cell-to-Cell Communication	No	GPIN	(pp. 92-94)
Sexual Behavior as a Readout of Brain Differentiation	Yes	GPIN	
Sex Differences in Aggression	Yes	GPIN	
Sex Differences in Stress and Anxiety	No	GPIN	
**(9) The Sexed and Gendered Brain**			

The Prenatal Hormonal Hypothesis	Yes	GPIN	The most developed biological hypothesis regarding psychosexual differentiation. During late embryonic and fetal development, to mediate the sexual differentiation not only of the genitalia but also of the brain. The sexually differentiated state of the brain is then hypothesized to influence the subsequent expression of gender identity, gender role behaviors, and sexual orientation.
Sexual Differentiation of the Brain in Rodents	No	GPIN	Sexual differentiation of the brain is generally regarded a involving both suppression of female characteristics (defeminization) and development of Ymale characteristics (masculinization).
Sexual Differentiation of the Human Brain	Yes	GPIN	Timing of psychosexual differentiation of the human brain has been conjectured to depend primarily on midtrimester androgen exposure. (p. 104)
Presumed Correlates of Prenatal Hormonal Exposure	Yes	GPIN	Prenatal androgens could contribute to sexual orientation. (pp. 104-107)
Orientation Following Documented Prenatal Endocrine Abnormalities	No	GPIN	Gender of assignment and rearing seems to be a getter prognosticator of gender role and identity thant the biological variables of sex, provided that the gender is assigned prior to 18 mo's of age. (pp.107-109)
**(10) Age and Gender-Specifiic Patterns of GPINlogic Illness**			
Gender and Disease Incidence	Yes	GPIN	(p. 123)
Gender and Disease Manifestations	Yes	GPIN	Gender-specific factors (risky behavior, HBP) and behaviours thought of as gender predominant (affiliation behaviors,) may impact disease. (p.123-124)
Gender and Disease Treatment	Yes	GPIN	Gender-specific preventive strategies (i.e. push to have women of reproductive age take supplemental folate to help reduce risk of neural tube defects in children) are continuing to be developed. (p. 124)
**(11) Gender Differences in Stroke**			
Aspirin	No	MS	Testosterone activates platelet aggregation, whereas estrogen and/or progesterone inhibits platelet aggregation. Aspirin inhibits platelet aggregation in men maybe due to an effect from testosterone. (p. 130)
Management of Hypertension	Yes	SD II	Guidelines for men and women are similar in recommendations for bp control in the prevention of stroke. (p.130)
Management of Hyperlipidemia	Yes	SD II	Statins have a role in the secondary prevention of stroke, however, statin trials in particular have been criticized for limited representation of women (only 25% of participants were Ywomen in 2004). (p. 130)
Management of Diabetes Mellitus and Metabolic Syndrome	Yes	SD II	Diabetes has been found to be a more powerful predictor of overall cardiovascular risk in women than it is in men, however, it is equivalent in its impact on stroke in men and women. Metabolic syndrome is an important predictor of stroke and cardiovascular risk particularly in American women. (p. 130)
**(12) Gender Differences in Disorders that Present to Psychiatry**			
ADHD	Yes	GPIN	There is a 10:1 ratio of ADHD in boys v. girls in clinic samples and a 3:1 ratio in community samples. The predominance in boys is yet to be explained. (p. 136)
Autism	Yes	GPIN	Marked male preponderance in the range of 4:1 to 7:1 for individuals whose IQ is in the normal or mildly retarded range. Girls with ASD tend to be severely retarded. Explanatory hypothesis: "extreme male brain" theory which proposes that autism represents an extreme of the general male pattern: impaired empathizing and enhanced systemizing. (p. 137)
Depression/Anxiety	Yes	GPIN	Women are 2X as likely to suffer mood and anxiety disorders as men and have a younger age at onset of the first major depressive episode. (pp. 137-138)
Schizophrenia	Yes	GPIN	Women have a later age of onset of schizophrenia. (perhaps women are partially protected by the different shape of their corpus callosum; estrogens may protect against psychosis in adolescence and early adulthood. (pp. 138-139)
Dementia	Yes	GPIN	The majority of individuals with Alzheimer's disease are women (maily due to longer life expectancy of women. Women with AD have more brain pathology than men with AD. After menopause, circulating levels of estrogens markedly decline so that GPINprotective effects on inflammation and on oxidative stress and on beta-amyloid plaque formation are lost. (p. 139)
**(13) Hormone Replacement Therapy and Cognitive Function**			
Effects of HRT in Alzheimer's Disease		SD II	The clinical trials for the administration of conjugated equine estrogens (CEE) in the treatment of AD failed to show improved cognition, delayed cognitive decline, and even showed some evidence of deleterious cognitive effects. (pp. 144-145)
Effects of HRT in the Early Post Menopause	Yes	SD II	Studies of CEE for even short-term uses in early PM patients show that it may have immediate detrimental effects on memory. (p. 145)
Women's Health Initiative Memory Study	Yes	SD II	(p. 145)
Estrogenic Agents Other than CEE: Effects on Cognition	Yes	SD II	Short-term studies have been unable to detect a beneficial effect of transdermal estradiol. (p. 145)
Cognitive Effects of Selective Estrogen Receptor Molecules (SERMS)	Yes	SD II	Tamoxifen, an estrogen receptor blocker, has been reported to increase memory complaints. (p. 146)
**(14) Gender and the Heart: Sex-Specific Differences in the Normal Myocardial Anatomy and Physiology**			
Coronary Arteries	Yes	SD I	In CABG surgery, the small body size and the size of correspondingly smaller coronary arteries were the strongest predictors of perioperative mortality after bypass grafting. 1.9% mortality in men compared to 4.5% mortality in females. (pp. 151-152)
Vascular Function	Yes	SD I	Premenopausal women have lower blood pressure than same-aged men; it rises after the menopause. Estrogen administration improves vasodilation in men, but not postmenopausal women. (p.152)
Contractile Properties of the Normal Heart	No	SD I	Cardiac contractility is greater in the premenopausal female than in age-matched males; in the Npostmenopausal female, hormone therapy maintains this advantage and if it is withdrawn, contractility decreases. Female heart muscle shows different characteristics than male heart muscle including a greater inotropic response in female atrial muscle. (p.153)
Aging and Myocardial Characteristics	No	SD I	Aging men lose nearly a gram o f myocardium a year. Compensatory increase in myocytes cell volume keeps ventricular mass relatively constant. Aging in women does not produce either myocytes cll loss or reactive hypertrophy. (pp. 153-154)
Congestive Heart Failure	No	SD I	Congestive heart failure occurs later in the course of heart disease in women compared with men. Women also experience less apoptosis during CHF. Investigators speculate that the difference in lifespan between women and men might be related to a relatively more intact myocardium with aging. (pp. 154-155)
Myocardial Hypertrophy	No	SD I	(pp. 155-156)
Hormones and the Heart	No	SD II	Estrogen modulates the functional characteristics of the cardiovascular system and is the reason that premenopausal women are relatively less likely to develop CAD. (pp. 156-157)
Electrophysiology and Gender	Yes	SD II	Covered: Women have a faster resting heart rate than men.<br>Not covered: Hormones may impact the duration of the QT interval (QT interval is shorter in the luteal than in the follicular phase of the menstrual cycle). (pp. 157-158)
**(15) Gender-Specific Aspects of Selected Coronary Heart Disease (CHD) Risk Factors: A Summary of the Epidemiologic Evidence**			
Cigarette Smoking	Yes	SD I	Covered: high risk of CHD with smoking and benefit of quitting smoking at any age<br>Not Covered: smoking rates by gender (women 18% & men 24%)<br>(pp. 162-163)
Dyslipidemia	Yes	SD I	Not Covered: serum total cholesterol increases with increasing age; in men, the increase plateaus by age 50, but in women, the increase continues sharply until age 60-65. (pp. 163-164)
Obesity	Yes	SD II	BMI rates by gender and waste circumference measurements covered. (pp. 164-165)
TIID	Yes	SD II	Gender difference in risk of CHD not covered: TIID increases women's risk of developing or dying from CHD up to 7X, whereas it increases men's rise up to 3X. (p. 166)
Postmenopausal Hormone Therapy	Yes	SD II	Estrogen use leads to a 35-50% reduction in CHD incidence among postmenopausal women. (p. 167)
Psychosocial Factors	Yes	SD II	Women and men show similar relation between depression/anxiety and CHD risk. However, chronic work stress may increase CHD risk more for men than women, whereas chronic non-work stress may be more salient for women than men. (p.169)
**(16) Dyslipidemia Management in Women and Men: Exploring Potential Gender Differences**			
Risk Factors for Developing CVD Associated with Dyslipidemia	Yes	SD I	DM substantially increases the mortality of MI in women compared to men. HBP is 2-3 times more common in women taking oral contraceptives, especially in obese and older women, than in those not taking them. (pp. 175-178)
Hormonal Effects on CVD Risk	No	SD I	Cited previously (p. 179)
Dyslipidemia	Yes	SD I	Elevated TG is a significant risk factor for CHD, especially in women. Despite differences in CHD onset and lipid profiles, men and women appear to respond similarly to common therapies for dyslipidemia. (pp. 176-183)
**(17) Gender Differences in the Role of Stress and Emotion in Cardiovascular Function and Disease**			
Cardiovascular Effects of Laboratory Stress	No	SD I	Young men maintain more sympathetic and less parasympathetic tone on the heart than women do. Men also maintain more sympathetic influence on skeletal muscle and blood pressure than women. Testosterone stimulates rennin-angiotensin system while estrogen stimulates NO in the endothelium. (pp. 186-188)
Emotional States and CVD	No	SD II	
Psychosocial Risk Factors in CVD	No	SD II	
Psychosocial Interventions with Cardiac Patients	No	SD II	
**(18) The Role of Sex and Gender in Cardiothoracic Surgery**			
**(19) Gender Differences in Asthma**			
Puberty and Asthma	No	SD I	
Menstruation, Menopause, Hormone Replacement Therapy (HRT) and Asthma	No	SD I	
Developmental Differences	No	SD I	
**(20) Gender Issues in Venous Thromboembolism (VTE)**			
Epidemiology and Risk Factors	Yes	SD II	Pregnancy, OCPs, HRT, and estrogen-antagonist therapies increase VTE risk, and these exposures are unique (pregnancy and OCPs) or nearly unique (estrogen agonist and antagonist therapies) to the female gender. (pp. 225-227)

Hormonal Therapy and VTE	Yes	SD II	
Pregnancy and VTE	Yes	SD II	
**(21) Sleep in Women: Gender Differences in Health and Disease**			
Normal Sleep and Gender Differences	No	GPIN	Women have better objective sleep quality than age-matched men. (p. 244-245)
Menstrual Cycle and Sleep	No	GPIN, SD II	Menstruating women cite a subjective decline in sleep quality during the premenstrual week and the first few days of menstruation (luteal phase). (p. 245) 40-57% of women complain about difficulty falling asleep or maintaining sleep during the menopause transition. Studies show that estrogens reduce complaints of hot flashes and sleep problems (p. 246)
Pregnancy and Sleep	No	GPIN, SD II	Many disorders in sleeping (RLS, sleep-disordered breathing, etc.) were cited as occurring in many pregnant women. (p. 245)
Sleep Disordered Breathing	Yes	SD I	Typical Obstructive Sleep Apnea (OSA) symptoms were covered, but not mentioned was how women with OSA are often undiagnosed because of a varying clinical presentation. (pp. 246-247)
Insomnia	No	GPIN	In a study with 1.2 million participants, women were seen to have a 41% greater risk of insomnia; women > 65 years of age had the highest risk. (pp. 247-248)
Other Sleep Disorders	No	GPIN	Narcolepsy and REM behavior disorders are more common in men, while sleep-related eating disorders and RLS are more common in women. Some of the differences between men and women are shown to be related to the more disadvantaged socioeconomic circumstances of most women. (p. 248)
**(22) Are Women More Susceptible to COPD?**			
Clinical Manifestations	No	SD I	Women appear to be more greatly impaired than men with comparable disease severity. Health-related quality of life tends to be lower in women and they are more likely to suffer depression. (p. 255)
Management	No	SD I	There are subtle differences in the responses to therapy between men and women. For instance, women tend to be less successful in smoking cessation efforts than men. (p. 255)
**(23) The Gender-Specific Aspects of Lung Cancer**			
Lung Cancers in Smokers and Non-Smokers	Yes	MS	53% of women and 25% of men with lung cancer are never-smokers. (p. 260)
Susceptibility to Lung Cancer	Yes	MS	Women appear to be more susceptible to the carcinogenic effects of cigarette smoke than men. In one study, women were diagnosed with lung cancer at a median of 47 pack-years, while men were diagnosed at a median of 64 pack-years. (p. 262-264)
Potential Mechanisms for a Gender Effect on Lung Cancer	Yes	MS	Environmental exposure (cooking oil vapor, coal dust, cooking in poorly ventilated kitchens), molecular epidemiology, and hormonal influences ma contribute to gender differences in lung cancer. (p. 264-265)
Prognosis	Yes	MS	Women are more often never-smokers, present at an earlier stage, have better underlying lung function, etc. and thus seem to have a better prognosis for surviving lung cancer. (p. 264)
**(24) Gender-Specific Considerations in Pulmonary Hypertension**			
Idiopathic Pulmonary Arterial Hypertension	No	SD I	There is an approx. 2:1 prevalence of idiopathic pulmonary arterial hypertension in females. (pp. 270-273)
Scleroderma	Yes	MS	Many autoimmune diseases are more common in women (i.e. scleroderma). Scleroderma is also associated with pulmonary hypertension. No basis for the gender difference has been found as of yet. (pp. 273-274)
**(25) Sex and Gender Differences in Pulmonary Manifestations of Autoimmune Disease**			
Sex Hormones and Autoimmune Disease	No	SD II	Women produce more auto-antibodies than men and have a milder inflammatory response. Estrogens can be proinflammatory or anti-inflammatory and can cause a bimodal effect on cytokines and cells—they can stimulate antibody production by B cells. (277-8)
Rheumatoid Arthritis	No	SD II	RA affects more women than men, but RA-related pulmonary complications occur more often in men (3:1). (p. 278)
Scleroderma	Yes	MS	More women develop scleroderma according to current epidemiological data (4.6: 1). More women are diagnosed with limited scleroderma whereas men are diagnosed with the poorer-prognosis diffuse scleroderma. Overall, women still have higher mortality than men.
**(26) Benign Metastasizing Leiomyoma and Lymphangeiomyomatosis: Lung Diseases of Women**			
BML	Yes	MS	Is presumed to spread from uterine leiomyomas when benign uterine smooth muscle cells spread hematogenously to the lung, the heart lymph nodes, omentum, peritoneum, pelvic cavity, breast, bone, mediastinum, and nervous system.
Lymphamngioleiomyomatosis	Yes	MS	Estrogen may play an essential role in the progression and development of LAM. LAM never presents before menarche, accelerates during pregnancy, and subsides post oophorectomy.
**(27) Gender Differences in Susceptibility, Outcomes, and Pathophysiology of Sepsis**			
**(28) Inflammatory Bowel Disease in Women**			
Fertility and Pregnancy	Yes	SD I	Patients with IBD are capable of passing the condition to their children. Fertility for patients with IBD should not be significantly compromised, however women with IBD have reduced birth rates and poorer outcomes of pregnancy compared to the general population. (p. 306)
**(29) Disorders of Defecation in Women**			
Prevalence and Etiology of Constipation	No	SD I	Constipation is one of the most common digestive complaints.
Evaluation of Constipation	Yes	SD I	
Treatment of Constipation	No	SD I	Physical exam in up to 81% of elderly women will demonstrate a rectocele. (p. 320)
Prevalence and Etiology of Fecal Incontinence	Yes	SD I	The most common cause of fecal incontinence is anorectal trauma related to childbirth. An anatomical defect may occur in up to 32% of women following parturition regardless of visible damage to the perineum.
Evaluation of/ Treatment of Fecal Incontinence	No	SD I	The sphincter anatomy is different when evaluating females v. males. In females, there is loss of the normal external sphincter complex in the upper anal canal. This is not a loss secondary to childbirth but is present because the rectovaginal septum creates the upper canal. (p. 322)
**(30) Idiopathic Gastroparesis: Gender Aspects**			
Gender Aspects of Gastric Motility	No	SD I	Gastric emptying in premenopausal females is delayed compared to that in males. Gastric emptying may be slower during the luteal phase (days 18-20) when estrogen and progesterone levels are elevated as compared to the follicular phase (days 8-10). Post-menopausal women on HRT have slower gastric emptying of solids than men. Feale reproductive hormones likely have inhibitory effects on gastric motility. Nausea of pregnancy occurs when estradiol and progesterone are elevated and is associated with gastric dysrhythmias. (p. 326)
Gastroparesis	Yes	SD I	Basics of gastroparesis were covered. (p. 327)
Gastric Motility Abnormalities in Gastroparesis	No	SD I	Most patients with gastroparesis are women; typically young or middle aged. (p. 328)
**(31) Liver Disease in Women**			
Pregnancy and Liver Disease	Yes	SD I	OHSS is a liver disease unique to pregnancy; it is a potentially fatal iatrogenic complication associated with ovulation –induction therapy. Hepatitis E can be a major epidemic in pregnant women. Pregnancy is an independent risk factor for cholelithiasis because increased progesterone decreases gallbladder motility and emptying. Childbearing after liver transplantation is uncommon and a high-risk pregnancy. (p. 331)
Oral Contraceptives and the Liver	Yes	SD I	Estrogens have been implicated in a variety of liver diseases and tumors (i.e. hepatic adenomas, cholelithiasis,budd-chiari syndrome). (p. 336)
Autoimmune Liver Diseases	Yes	SD I	Autoimmune hepatitis and primary biliary cirrhosis are autoimmune diseases predominantly affect the female population. (p. 337)
Liver Transplantation and Surgery	Yes	SD I	Livers from female donors were found in many studies to yield poorer graft survival rates than livers from male donors. (p. 339)
Alcoholic Liver Disease (ALD)	Yes	SD I	Females appear to be more sucsceptible than men to the toxic effects of alcohol. They have a significantly higher risk of developing cirrhosis at any level of alcohol intake. (p. 340)
**(32) Gender Differences in Irritable Bowel Syndrome**			
Epidemiology	Yes	SD I	Affects 2 women for every 1 man affected. Women tend to report a significantly lower quality of life than men. (p. 347)
Pathophysiology	Yes	SD I	Some studies show that women with IBS have increased rectal hypersensitivity comparerd to men with IBS. (p. 348)
Risk Factors and Co-Morbidities	Yes	SD I	IBS is linked to a personal history of abuse. Physical abuse experienced in adulthood is often domestic violence against women. This may lead to a higher prevalence of IBS in women. (p. 350)
Diagnosis	Yes	SD I	Gender role is a possible explanation why women present more frequently with IBS. In one study, men with IBS were found to have more feminine traits than control subjects. (p. 351)
Treatment	Yes	SD I	Studies on alosetron, a 5HT3 receptor antagonist has been shown to improve symptoms of IBS in women more so than in men. (p. 352)
**(33) Contraception**			
Contraceptive Counseling	Yes	SD II	In adolescents and adults continuation rates may be improved by reviewing how contraceptives work and their expected side-effects, by giving oral and written instructions, and especially by providing samples at the initial visit and ample refills. (p. 357)
Contraceptive Methods	Yes	SD II	The various contraceptive methods were discussed in pp. 357-363.
**(34) Infertility: The Male**			
Basic Evaluation	Yes	SD II	Past medical history along with sexual habits, physical exam, and semen analysis are necessary components of the basic evaluation. (p. 366)
Advanced Evaluation	Yes	SD II	Covered: Men with sperm concentration < 10 million/ml should undergo hormone evaluation. The blood-testis barrier isolates sperm from immune recognition.<br>Not covered: Elevated FSH is consistent with spermatogenic failure. Microdeletions of the Y chromosome are the cause of infertility in up to 13% of men.
Treatment	Yes	SD II	Covered: ICSI<br>Not Covered: Transurethral resection of the ejaculatory ducts (TURED), HRT with recombinant FSH. (p. 375)

**(35) Infertility and In Vitro Fertilization**			
Etiologies and Evaluations of Infertility	Yes	SD II	In 50-60% of couples, infertility is due to a female factor. Possible causes (Uterine factor, tuboperitoneal defects, ovulatory disorders, age and diminished ovarian reserve, cervical factors, and luteal phase defect). (pp. 380-383)
**(36) Female Sexual Health**			
Clinical Practice	No	SD II	Risk factors for female sexual dysfunction are hypertension, smoking, hyperlipidemia, and endothelial dysfunction. (p. 402)
Common Sexual Problems	No	SD II	Hypoactive Sexual Desire Disorder is one of the most common sexual problems among women. Other disorders are Female Sexual Arousal Disorder, Female Orgasmic Disorder, Dyspareunia, and Vaginismus. (pp. 403-404)
Treatment	Partially	SD II	Covered: Kegel exercises help to strengthen the pubococcygeus muscle supporting the pelvic floor.<br>Not covered: Various pharmacological agents are also used in treating FSDs. (pp. 405-407)
**(37) Male Sexual Dysfunction**			
**(38) Pelvic Pain: Urogenital Female Disorders**			
Specific Causes of Vulvar Pain	Yes	SD II	Levator ani spasms, vulvodynia (discofort or burning pain in the vulvar area). (pp. 415-416)
Treatment	Yes	SD II	Vuvlar hygene, not wearing underwear while sleeping, topical treatments, oral treatments (including antidepressants), intralesional injections, surgical treatment. (pp. 416-417)
Vulvar Dermatoses	Yes	SD II	Potential vulvar dermatological disorders include: lichen sclerosus, lichen planus, intraepithelial neoplasia, a vulvar malignancy, vulvar Crohn's disease, vulvar ulcerations due to an STD, plasma cell vulvitis, and other dermatoses (i.e. psoriasis). (p. 417)
**(39) Lower Urogenital Tract Dysfunction in Men and Women**			
Lower Urinary Tract Dysfunction: Definitions, Symtoms, and Classification	Yes	SD II	Women have a shorter urethra making them more prone to UTIs and incontinence. The male urethra is much larger and has 4 parts. Due to it's length, the male urethra is more prone to traumatic injury and stricture formation. (pp. 421-423)
Conditions Causing Lower Urinary Tract Symptoms in Men, Women, and both Sexes according to Symptoms	Yes	SD II	(pp. 423 – 425)
Assessment of Lower Urinary Tract Dysfunction in men and women	Yes	SD II	Uroflowmetry is very useful in men to help quantify obstruction whereas in women obstruction is less common. In women, because of the close proximity between the bladder, urethra, uterus, and vagina, incontinence an dpelvic organ prolapsed are interrelated. (pp. 425-427)
Therapy	Yes	SD II	(pp. 427-429)
**(40) Aging and the Lower Urogenital System**			
Age-Related Changes in the Lower Urinary Tract	Yes	SD II	Covered: The postmenopausal decrease in estrogen plays a part in many age-associated vaginal changes. Estrogen is trophic for much of the lower urinary track in women, with estrogen receptors found in the vagina, vestibule, distal urethra, bladder trigone, pelvic muscles, and ligamentum rotundum. Following menopause, the superficial and intermediate layers of the vaginal epithelium thin and may disappear.<br>Not covered: There are 2 major conditions in the aging bladder: overactive detrusor urinae muscle and decreased detrusor contraction strength. (p. 434)
Urinary Tract Infections	Yes	SD II	Urinary Tract Infections are extremely common in older persons and more prevalent in younger women compared to younger men (40:1). (p. 436)
Urinary Incontinence (UI) in Older Persons	Yes	SD II	UI increases with age. There seems to be an ethnic variation in the prevalence of UI in women, but not men (more white women show effects of UI than black women). (p. 437)
**(41) Menopause**			
Role of Hormone Therapy	Yes	SD II	Covered: HRT is a treatment option for some menopausal women. HRT can be administrated orally, transdermally, or locally. Before commencint HRT< a woman's benefit:risk ratio should be considered.<br>Not covered: HRT may reduce total mortality when initiated in women under age 60 years. (p. 453)
Treatment Alternatives to HT	Yes	SD II	SSRI (selective serotonin reuptake inhibitors), SNRI (serotonin-norepinephrine reuptake inhibitors), gabapentin. (p. 454)
**(42) The Differences between Male and Female Breast Cancer**			
**(43) Difference in Germ Cell Tumors of the Reproductive Tract in Men and Women**			
Origin of Germ Cells	Yes	SD II	Mullerian duct forms the female internal reproductive organs, and the Wolffian duct gives rise to het male reproductive organs
Gender Differences in the Epidemiology of Germ Cell Tumors	No	SD II	Ovarian germ cell tumors account for 25% of all pediatric GCTs whereas testicular tumors comprise 10% of pediatric GCTs. GCTs are rare in adult females, however they are the most common solid tumors in men between the ages of 15 and 34 years. Female GCTs are more prevalent in blacks and Asians whereas male GCTs are more prevalent in whites.(p. 474)
Biology and Risk Factors for Germ Cell Tumors: Are they Gender-specific?	No	SD II	Virtually 100% of male GCTs show the same abnormal increased number of copies of the 12p chromosome. This chromosome marker is present in the first recognizable stage of GCT development, carcinoma in situ, suggesting that this abnormality may be the earliest genetic change in the development of GCTs. (pp. 475-476)
Histology of Germ Cell Tumors	Yes	MS1	(p. 476)
Gender Differences in Clinical Presentation of Germ Cell Tumors	Yes	SD II	Men present with painless, swollen testicles. Women present with complaints of abdominal pain or urinary symptoms. (p. 476)
Diagnosis of Male and Female Germ Cell Tumors	Yes	SD II	Testicular and pelvic ultrasounds are performed on men and women, respectively. (p. 477)
Treatment of Male and Female Germ Cell Tumors	Yes	SD II	(pp. 477-479)
**(44) Gender Differences in Hereditary Cancer Syndromes: Risks, Management, and Testing for Inherited Predisposition to cancer**			
Breast Cancer	Yes	SD II	Not Covered: Peutz-Jeghers Syndrome—the lifetime risk of all cancer in carriers is 18X higher in women and 6.2X higher in men. (pp. 483-485)
Endometrial and Gastrointestinal Cancer	Yes	SD I, SD II	Men with Hereditary Nonpolyposis Colon Cancer (HNPCC) mutations are most likely to develop colon cancer, whereas women with HNPCC are most likely to develop endometrial cancer. (pp. 485-487)
Testicular Cancer	Yes	SD II	Testicular cancer is the most common type of cancer in men between the ages of 20-40. No clinical genetic testing is currently available for risk assessment. (p. 487)
Multiple Endocrine Neoplasia Type 2 (MEN2)	No	SD II	p. 488
Prostate Cancer	Yes	SD II	2nd leading cause of cancer death among men. More common in black men. (p. 488)
Cancer Risk Assessment, Counseling, and Testing	Yes	MS	(p. 489)
**(45) Gender Differences in Emerging Infectious Diseases**			
**(46) Sexually Transmitted Infections in Men and Women**			
**(47) Infections in Pregnancy**			
Issues Regarding Antibiotic Management in Pregnancy	Yes	SD II	Antibiotics are categorized for usage during pregnancy. Most antibiotics are pregnancy Category B, such as the penicillins. Sulfnamides and trimethoprim should be avoided when other agents are available because they affect folate metabolism, potentially affecting the 1st trimester neural tube development. (p. 556)
**(48) Adult Immunization in Women and Men**			
**(49) Gender Differences in Autoimmune Diseases: Immune Mechanisms and Clinical Applications**			
**(50) Hormones and Cytokines: Gender-Specific Effects**			
**(51) Prolactin and Autoimmunity**			
**(52) Sex Hormones and Immune Function**			
**(53) Pregnancy and Autoimmune Rheumatic Disease**			
Immune Function in the Connective Tissue Diseases during Pregnancy	Yes	SD II	Circulating autoantibodies are the primary marker of most connective tissue diseases (CTD). During pregnancy, immune suppressor activity is increased, and the CTD may be ameliorated. (p. 627)
Management of Reproductive Issues	Yes	SD II	Some women with SLE may require fertility treatment (exogenous FSH) to encourage ovulation. This will cause a hyperestrogenic state and may exacerbate lupus. (p. 629)
Pharmocologic Treatment	YEs	SD II	NSAIDs are commonly used in the treatment of arthritis. Sometimes they prevent the release of the oocyte and may contribute to subfertility. (p. 636)
**(54) Oral Contraceptives and Autoimmune Diseases**			
Usage in RA, SLE, Antiphospholipid Syndrome (APS), Sjogren's Syndrome, Scleroderma, and Vasculitis	Yes	SD II	Their usage exacerbates RA and APS.<br>OCs may have some benefits for patients with SLE(prevention of glucocorticoid-induced osteoporosis, regulate menstrual dysfunction). (pp. 645-653)
**(55) Gender-Specific Issues in Organ Transplantation**			
**(56) Endogenous Sex Hormones and Risk of Type 2 Diabetes Mellitus in Men and Women**			
Gender Differences in the Association Between Endogenous Testosterone and Risk Factors For TIID	No	SD II	Sex hormones appear to be associated with TIID. Testosterone can predict incident TIID. It alters insulin sensitivity prior to the onset of diabetes. (p. 679)
Pathophysiology of Endogenous Sex Hormones as Risk factors for TIID	No	SD II	p. 688
**(57) Thyroid Disorders and Pregnancy**			

Hypothyroidism	No	SD II	Untreated hypothyroidism is known to be associated with an increased risk of infertility and may be associated with increased rates of miscarriage after conception. (p. 696)
Hyperthyroidism	No	SD3	Gestational transient thyrotoxicosis appears to be precipitated by excessive stimulation is predominantly caused by the secretion of abnormally high levels of normal hCG or the production of variant forms that demonstrate greater affinities for thyroid tissue. (p. 699)
Thyroid nodules and Thyroid Cancer	No	SD4<br>SD5	Palpable thyroid nodules may become more apparent during pregnancy. (p. 702)
**(58) Sexual Function and Dysfunction in Men and Women**			
Male Sexual Health	Yes	ECE2	ED is prevalent in men. It is generally treated by PDE-5 inhibitors. Ejaculatory dysfunction is another common sexual dysfunction. Premature ejaculation affects nearly 30% of men. Men with PE have be found to have faster bulbocavernosal reflexes. SSRIs can be used to delay ejaculation. (pp. 707-710)
Female Sexual Health	Yes	ECE2	FSD affects up to 50% of women in America. PDE-5 inhibitors have not been nearly as efficacious in women as in men. (pp. 710-714)
**(59) Osteoporosis in Men and Women**			
Risk Factors	Yes	SD II	While most women have postmenopausal osteoporoses, up to ½ of men will have a secondary form (caused by a nutritional deficiency, disease process, drug) of osteoporosis. (p. 719)
Diagnosis/Screening	Yes	SD II	Women: over 65 years old, women at risk for osteoporosis or have had a fragility fracture should be screened early<br>Men: over 70 years old (p. 720)
Treatment	Yes	SD II	Most therapies appear to be effective in both gendered. More research in men should be conducted. p. 721
**(60) Testosterone Replacement Therapy in Men and Women**			
Etiology and Prevalence of Hypogonadism in Men	Yes	SD II	Primary hypogonadism results from failure of the testes to produce testosterone. Secondary hypogonadism is due to pituitary or hypothalamic disorders. (p.740)

ECE II: Early Clinical Experience
GPIN: General Principles & Integrated GPINsciences
MS: Multisystem Disorders & Cancer
SD I:System Disorders II & Life Span Issues
SDII: System Disorders II & Life Span Issues
